# Comparison of the extractability of organophosphorus flame retardants in landfill media using organic and green solvents

**DOI:** 10.1038/s41598-022-13704-1

**Published:** 2022-06-09

**Authors:** Innocentia Velaphi Sibiya, Okechukwu Jonathan Okonkwo

**Affiliations:** Department of Environmental, Water and Earth Sciences, Tshwane University of Technology, Tshwane, South Africa

**Keywords:** Environmental sciences, Environmental chemistry, Environmental monitoring, Green chemistry

## Abstract

Organic solvents are mainly used in the extraction of organophosphorus flame retardants (OPFRs) because of their availability and having been tested as good extracting solvents for most environmental pollutants. However, organic solvents are toxic, flammable, and costly. Hence, there is an ongoing quest for less hazardous chemicals such as green deep eutectic solvents (DES) that are cheap, recyclable, non-toxic and degradable in the environment, which can be used to extract organic pollutants such as OPFRs in environmental samples. This study assessed the extractability of OPFRs in municipal landfill leachate and sediment, using organic solvents and DES. Of the fourteen targeted OPFRs, 11 (80%) and 7 (50%) were detected in the leachate and sediment samples, using hexane; whereas 14 (100%) and 13 (90%) OPFRs were detected in the same order of samples using DES. The concentrations of OPFRs obtained for the leachate using optimum organic and DES ranged from below the limit of quantification (< LOQ)—516 ± 8.10 ng/L and < LOQ—453 ± 8.10 ng/L respectively. Correspondingly, the concentrations of OPFRs in sediment samples ranged from < LOQ—135 ± 2.89 ng/g dw and < LOQ—395 ± 2.24 ng/g dw, respectively. The results from this study, therefore, highlight the potential of DES to extract more OPFR from complex matrices such as landfill leachate and sediment. This finding infers that green hydrophilic DES can serve as good replacement for organic solvents such as hexane in liquid–liquid extraction (LLE) and solid–liquid extraction (SLE) techniques for landfill leachate and sediment.

## Introduction

Flame retardants are organic chemicals that are added to materials, to reduce the rate at which materials catch fire, thereby giving people more time to escape^1^. With the gradual discontinuation of the use of flame retardants such as polybrominated diphenyl ethers (PBDEs), the demand for replacements increased and organophosphorus flame retardants were introduced as suitable replacements. However, research on these chemicals has re-emerged as they have been identified as not being quite suitable substitutes for the phased-out commercial penta- and octa-BDE formulations because of their toxicity^2^. Due to the application of organophosphorus flame retardants (OPFRs) in several household and industrial products, these products are the primary sources of OPFRs^3,4^. Hence, they can reach the environment via industrial emissions during manufacturing, and by leaching out of OPFRs-treated materials into the environment when disposed into municipal landfill sites^5^. In South Africa, consumer products inclusive of OPFRs-treated products are usually disposed of into landfill sites as general waste at the end of their life cycle. It is, therefore, important to monitor the presence of OPFRs in the landfill environment since groundwater contamination is most likely to occur also if no geomembrane liner is provided in the landfill^6^.

OPFRs have a wide range of polarities, and the reported studies in landfill leachate and water samples have used organic solvents that are polar^7–10^; a mixture of equal ratios of polar and non-polar organic solvents^11–14^; and unequal variations of polarities^13,15,16^. Only a few studies have reported the extraction of OPFRs from landfill sediment or soil samples, however, the use of polar^8,17,18^; and unequal variations of organic solvents were reported^14,15,19^. The use of organic solvents for extraction is owed to their availability and having been tested as good extracting solvents for most organic environmental pollutants^20^. However, it is a well-known fact that these solvents are toxic, flammable, and costly^21^. Hence, there is an ongoing quest for less hazardous chemicals with a high extracting potential that can be used to extract organic pollutants such as OPFRs in environmental samples.

Several studies have reported the use of deep eutectic solvents to extract flame retardants or pesticides in environmental matrices. For example, Solaesa et al.^22^ reported the extraction of PBDEs in fish oils using choline chloride: phenol (1:2) deep eutectic solvent (DES); and a study by Shahbodaghi, et al.^23^ reported the extraction of OPFRs in tap water, wastewater, river water and well water using benzyltriphenylphosphonium bromide (BTPPB): 2-dodecanol as DES. Yousefi, et al.^24^ used choline chloride:urea (1:2) DES to extract hexachlorocyclohexane (HCHs (α-, β- and γ)), heptachlor, aldrin, heptachlor-endo-epoxide, α-endosulfan, dieldrin, endrin, betaendosulfan, endrinaldehyde, endosulfan-sulfate, dichlorodiphenyltrichloroethane (DDT) metabolites (pp-DDE, pp-DDD and pp-DDT), endrin-ketone and methoxychlor pesticides in farmwater, rural water, lakewater and river water. All the above mentioned studies used hydrophobic DES, with the exception of the latter study on pesticides. No information was found on the extraction of OPFR compounds in environmental aqueous media, more so in sediment or soil media using hydrophilic choline-based DES. DES are inexpensive to prepare, thermally stable, have extremely low toxicity, and are biodegradable.

Hence, in this study, owing to observed variations from previous studies using organic solvents, first liquid–liquid extraction (LLE) and solid–liquid extraction (SLE) was optimised for the extraction of OPFRs in landfill leachate and sediment. Second, hydrophilic green deep eutectic solvents (DES), were synthesised, and employed in optimising LLE and SLE for the extraction of OPFRs in landfill leachate and sediment. The extractability of the optimum organic and green DES solvents using optimal conditions was validated, applied, and the data compared. Landfill leachate and sediment extraction solvents with better OPFR extractability are recommended.

## Materials and methods

### Chemicals and materials

Details of this section can be found in Supplementary Text S1.

### Study area

**Figure 1:**
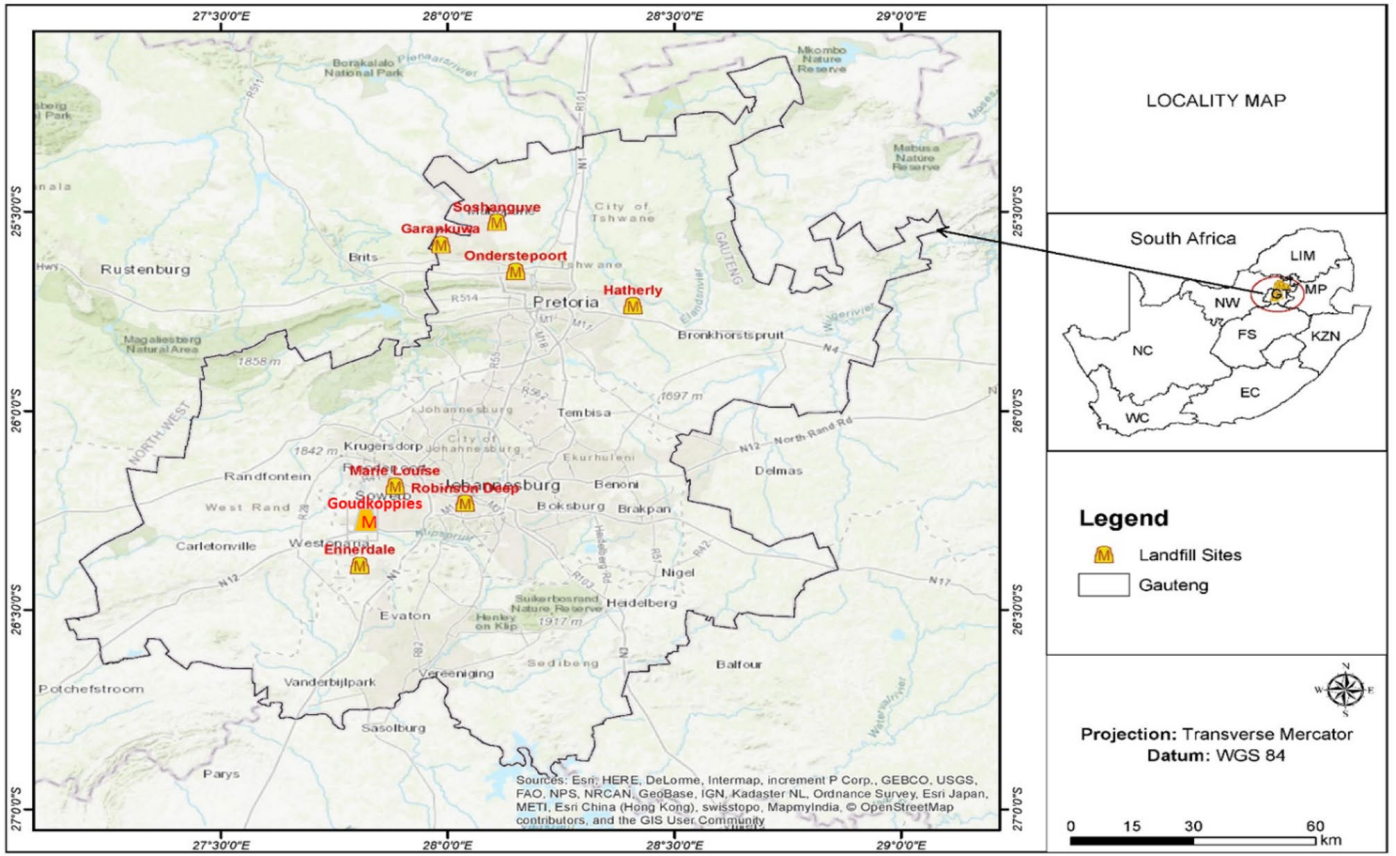
Map of South Africa (top right) and Gauteng Province (centre) showing the selected landfill sites. [The figure was generated by Sogayise^25^ in ESRI's ArcGIS Version 10.1, by using ArcMap with a “World Topographic Map” as a background (http://www.arcgis.com/home/item.html?id=30e5fe3149c34df1ba922e6f5bbf808f)].

Landfill leachate and sediment samples were collected from selected landfill sites in the cities of Tshwane and Johannesburg, Gauteng Province, South Africa. More details about the study area are as described by Sibiya, et al.^15^. Samples were collected from eight sampling sites in June 2019, as shown in Fig. 1. June is a winter month in the southern hemisphere. The selected municipal landfills were Hatherly, Soshanguve, Onderstepoort and Ga-Rankuwa in the City of Tshwane, and Ennerdale, Goudkoppies, Marie Louise and Robinson Deep in the City of Johannesburg.

### Sample collection and preparation

The raw leachate (2 L each) and sediment (1000 g) samples were collected in triplicates on pre-washed and acetone-rinsed amber bottles during the winter of 2019. Leachate samples were collected from the leachate ponds using the grab sampling method and sediment samples were collected between 0 and 5 cm below the surface of the leachate retention pool from the same location as the leachate sample. In one site at the leachate pond, the leachate and sediment samples were taken from the North, South, and Central side of the pond. A more detailed description of the sampling method can be found in the article by Sibiya et al.^15^. The samples were covered immediately after sampling, stored in cooler boxes, transported to the laboratory, and kept at − 4 °C in a cold room. The collected sediment samples were air dried under the fume hood at room temperature (25 °C). Subsequently, they were homogenised using a pestle and mortar and sieved (150 µm) to remove wood splinters, glass, and stones.

### Instrumentation

Liquid chromatography- tandem mass spectrometry (LC–MS/MS) was used to quantify the targeted OPFRs. The conditions and procedure used can be found in Supplementary Text S2, and retention times, precursor (Q) and product ions (q) of OPFRs in Supplementary Table S1.

### DES synthesis

The DES were prepared by heating the hydrogen bond acceptor (HBA) : hydrogen bond donor (HBD) mixtures with constant stirring until a homogeneous liquid was formed, as shown in Table 1. Full details of the synthesis are provided in Supplementary Text S3. More viscous DES, such as choline chloride/zinc choride (1:2) (DES-2), choline chloride/oxalic acid dihydrate (1:2) (DES-4) and choline chloride/D-Fructose (5:2) (DES-6), were further diluted with different fractions of water before extracting OPFRs from sediment as can be seen in Supplementary Table S2. Information on how the DES was charecterised can be found on Supplementary Table S3.

**Table 1:** Deep eutectic solvents’ preparation conditions.

DES	HBA	HBD	Molar ratio	Synthesis temperature (°C)
DES-1	ChCl	Urea	1:2	80
DES-2	ChCl	ZnCl_2_	1:2	110
DES-3	ChCl	OxAc	1:1	90
DES-4	ChCl	OxAc	1:2	90
DES-5	ChCl	OxAc	2:1	90
DES-6	ChCl	D-Fructose	CD (5:2)	150

ChCl = choline chloride, OxAc = Oxalic acid dihydrate, DES-1 = ChCl:Urea (1:2), DES-2 = ChCl:ZnCl_2,_ DES-3 = ChCl:OxAc (1:1), DES-4 = ChCl:OxAc (1:2), DES-5 = ChCl:OxAc (2:1) and DES-6 = ChCl:D-Fructose (5:2).

### Optimisation of organic solvents and DES for the extraction of OPFRs in the leachate and sediment

Liquid–liquid extraction (LLE) and solid–liquid extraction (SLE) were used in extracting OPFRs from the leachate and sediment samples. Organic solvents, ethyl acetate, hexane: dichloromethane (4:1); hexane: acetone (3:1), hexane: ethyl acetate (1:1) and hexane; and green choline/chloride urea (1:2) (DES-1) to DES-6 were tested for their extractability of OPFRs in landfill leachate and sediment. The solvents were tested under the following extraction parameters: sample volumes (5, 10, 15, 20 and 25 mL), masses (0.3, 0.5, 1, 1.5 and 2 g), sonication time (5, 10, 15 and 20 min), vortex time (30, 60 and 120 s), centrifuge speed (2500, 3000, 3500 and 4000 RPM) and centrifuge time (5, 10, 15 and 20 min) (see Supplementary Fig S1, Fig S2, Fig S3 and Fig S4).

A full description of the optimisation procedures can be found in Supplementary Text S4, and Supplementary Fig S5 is a schematic representation of the sample clean-up procedure. The optimum extracting parameters obtained for hexane, choline chloride/oxalic acid dihydrate (1:1) (DES-3) and DES-1 is summarised in Table 2. Supplementary Fig S6 and Fig S7 show the extraction flow chart of the application procedure of the targeted OPFRs in the leachate and sediment, respectively using green DES solvents (DES-3 and DES-1) that were optimum for extraction. Supplementary Text S5 and S6 explain the validation and application protocols depicted by the flow charts for DES; and Supplementary Text S7 explains protocols using hexane for extraction.

**Table 2:** Optimum OPFR extraction conditions obtained for the leachate and sediment using hexane and green DES solvents.

Extraction parameter	Leachate		Sediment	
	Organic solvents	DES solvent	Organic solvents	DES solvent
Sample volume (mL)/mass (g)	5	5	1	0.75
Extraction solvent (mL)	Hex	DES-3	Hex	DES-1
Sonication time (min)	20	5	10	10
Vortex time (min)	1	1	1	1
Centrifuge speed (RPM)	3500	3500	4000	2500
Centrifuge time (min)	20	5	5	5

Hex = hexane; DES-3 = choline chloride/oxalic acid dihydrate (1:1) and DES-1 = choline chloride/urea (1:2).

### Quality assurace (QA)/quality control (QC)

An internal standard (IS) mix of triphenyl phosphate-d_15_ (dTPP), tributyl phosphate-d_27_ (dTBP), tri-n-propyl phosphate-d_21_ (dTPrP) and ^13^C_18_-triphenyl phosphate (^13^C_18_MTPP) was used for spiking one municipal (Hatherly) and one industrial (Marie Louise) landfill leachate and sediment during optimisation. Spiked deionised water for the leachate and sodium sulphate for sediment was also used as a part of the method optimisation process. The latter are referred to as quality control (QC) blanks in this study (see Supplementary Text S4). To validate the extraction method, EDF-2525 contaminated natural matrix reference material previously used in the first worldwide inter-laboratory study on OPFRs^26^ was applied to the optimum sediment extraction method using hexane and DES-1. The OPFR compounds in the certified reference material (CRM) are the neat targeted compounds, hence the CRM was used for method validation. The neat OPFR standards were not used for spiking during method development, since they may be present in landfill leachate and sediment^15^. Measured CRM values were within the certified range of ± 20% (see Supplementary Table S4 and S5). To validate the leachate extraction, the spiking method was used with DES-3 as the extracting solvent. LOQs ranged from 0.001 to 0.01 ng/L in the leachate and 0.08–0.12 ng/g dw in sediment for hexane; 0.01–5.00 ng/L in the leachate for DES-3; and 0.10–10.0 in sediment for DES-1. LOQs were determined as described in Supplementary Text S8.

## Results and discussion

### Chemical properties of DES

Fourier Transform Infrared (FTIR): Supplementray Figs S8–S11 show FTIR spectra for four DES and their starting materials with key functional groups of the compounds involved in the synthesis of DES. Choline chloride shows vibrational bands at 3.20 × 10^3^–3.55 × 10^3^ cm^−1^, which refers to hydroxyl functional groups (-OH). Vibrational bands at 2.85 × 10^3^ cm^−1^ and 3.01 × 10^3^–3.10 × 10^3^ cm^−1^ refer to alky groups -CH_2_ and -CH. The tertiary amine peaks were not observed in the choline chloride spectra. Most of the functional groups observed on the pure choline chloride spectra were not observed after the synthesis of the DES^27^.

Supplementary Fig S8 shows the spectrum of DES-1. Urea exhibited two vibrations of primary amines (N–H stretching absorptions) at 3.42 × 10^3^ cm^−1^ and 3.33 × 10^3^ cm^−1^. It also shows amide with a, C=O stretch featuring between 1.69 × 10^3^ and 1.63 × 10^3^ cm^−1^. The DES-1 spectra shows vibrations that are similar to the urea spectra but these are generally more suppressed^28^.

The spectra of zinc chloride (see Supplementary Fig S9) shows vibrational frequencies at 3.46 × 10^3^ cm^−1^ (broad band) and 1.60 × 10^3^ cm^−1^ (sharp small band) which could be due to the compound absorbing of moisture. The resultant DES-2 is also characterised by the same moisture peaks at 3.46 × 10^3^ cm^−1^ and 1.62 × 10^3^ cm^−1^^29^.

Supplementary Fig S10 is the spectra of DES-3; oxalic acid dihydrate–OH stretches between 3.50 × 10^3^–3.20 × 10^3^ cm^−1^, carbonyl (C=O) stretches from the carboxylic acid^30^. DES-3 also has functional groups that are like the oxalic acid spectra, but these are less pronounced. The spectra for DES-4 is not shown since it showed similar vibrations and stretches as the DES-3 except more pronounced oxalic acid peaks were observed compared to the 1:1 ratio. Also, when the ratio is (2:1) (DES-5) an alkyl (C-H) bend was observed at vibration 2.56 × 10^3^ cm^−1^ and a C=O stretch at a vibration range 1.61 × 10^3^–1.80 × 10^3^ cm^−1^.

D-fructose (see Supplementary Fig S11) showed -OH bending bands at 3.51 × 10^3^ cm^−1^ and 3.38 × 10^3^ cm^−1^, scissoring –CH and CH_2_ bands at 3.10 × 10^3^ cm^−1^ and 2.89 × 10^3^ cm^−1^. The synthesised DES-6 unlike the preceding DES studied, showed functional groups and vibrations similar to choline chloride. It exhibited –OH bands at 3.46 × 10^3^ cm^−1^ and 3.22 × 10^3^ cm^−1^ vibrations, –CH and –CH_2_ bands at 3.01 × 103 cm^−1^ and 2.85 × 103 cm^−1^ vibrations.

Proton (^1^H) NMR: The chemical structure of the synthesised DES was confirmed by NMR spectroscopy. DES were diluted with hexadeuterated dimethyl sulfoxide (DMSO-d_6_) without further pre-treatment. DES are formed by hydrogen bonding by a halide ion and a hydrogen donor responsible for the low melting point of the mixture. Due to the hydrogen bonding interactions of the solvents, they can be well characterised by ^1^H nuclear magnetic resonance (NMR)^31^. As shown in Supplementary Fig S12–S15^1^H NMR spectra of DES from the four main families (DES-1, DES-2, DES-3 and DES-6) selected in this study are listed. In Supplementary Fig S12, it can be observed that after synthesis, all the protons from the starting contituents were in the synthesised DES. More so, these protons had noticeable upfield shifts when compared to those in their starting constituents. This response could be due to the charge delocalisation that occurred through hydrogen bonding of the starting materials that resulted in lower melting points relative to the melting points of individual components of the DES^27^. In Supplementary Fig S13 as expected, no protons were observed for zinc chloride except the deuterated chlororform and the DMSO-d_6_ peaks were present. All the protons found in choline chloride were also found in DES-2, with the upfield shifts of all protons from choline chloride (a) observed in the final DES-2 (c). For DES-3 in Supplementary Fig S14, it can be observed that the 1H proton that is downfield of the choline chloride (a) was involved in a proton exhange due to the diminishing signal (1H) on the synthesised DES (c). The broad 6H observed on the oxalic acid dihydrate (b) (1:1) could be due to the additional 4Hs from the two water molecules (dihydrate) of the oxalic acid (2H), resulting in 6H with no neighboring protons, resulting in the observed broad peak. The spectra for ratios 1:2 (DES-4) and 2:1 (DES-5) are not shown because they show similar peaks as the DES-3 spectra, the difference is that the number of protons and peak intensity increase with the increase in the ratio of either HBA or HBD constituent. The hydrogen deficiency index (HDI) of D-Fructose ^1^H NMR spectra (see Supplementary Fig S15) could not be calculated, even after being diluted as a result of the cloudy nature of the solution. However, ^1^H NMR was successfully used to confirm the structures of the starting materials, and the DES formed. Downfield to upfield chemical shifts (ppm) of protons proved that eutectic mixtures in which hydrogen is donated were formed.

### LLE and SLE optimisation with organic and choline-based DES for extraction

Effect of sample volume and mass: The effects of sample volumes were evaluated, hexane:acetone (3:1) and DES-3 were used to begin the optimisation process for the leachate samples. Hexane:acetone (3:1) was selected based on its effiency to extract OPFRs as reported by Sibiya et al.^15^. DES-3 was selected due to its less viscous nature and ability to efficiently extract polycylic aromatic hydrocarbons (PAHs) using LLE (Mbous et al., 2016; Płotka-Wasylka et al., 2017). The highest recoveries of internal standards (IS) were obtained at 5 mL sample volume ranging between ^13^C_18_ MTPP (71.6 ± 7.62%) in Marie Louise and dTPP (103 ± 11.4%) in Hatherly with organic solvents (see Supplementary Fig S3(b)). Using DES, the highest recoveries of IS were also obtained at 5 mL sample volume ranging between dTBP (90.4 ± 2.21%) and dTPrP (102 ± 2.13%) in Marie Louise and dTPrP (92.8 ± 4.62%)–dTPP (99.7 ± 9.23%) in Hatherly (see Supplementary Fig S1(b)). Five millitres was used as the optimum extraction sample volume thereafter.

Supplementary Fig S4(a) and Fig S2(a) shows the effect of the sample mass, using the same organic and DES solvents used with the leachate samples to kickstart the optimisation; similarly the IS used for spiking the leachate was also used for sediment samples. The highest recoveries of the spiking IS in all the samples were observed on the 1 g sample mass ranging from dTPP (96.0 ± 8.52%) in Hatherly to dTPrP (109 ± 3.45%) in Marie Louise with organic solvents. With DES, the highest recoveries of the spiking IS in all the samples were observed on the 0.75 g sample mass ranging from dTPP (95.8 ± 8.24%) in Hatherly to ^13^C_18_MTPP (108 ± 5.03%) in Marie Louise. Hence, moving forward 1 g for organic solvents and 0.75 g for DES was used as the optimum masses for extraction.

Effect of extraction solvent: Five millitres of the selected organic solvents was used to extract 5 mL of the leachate and 1 g of sediment samples and the procedure was repeated twice on the same sample resulting in 10 mL of the extraction solvent (see Supplementray Fig S3(b) and Fig S4(b)). The highest recovery rates of IS were obtained using hexane as the extraction solvent ranging from ^13^C_18_MTPP (76.6 ± 8.88%) to dTPP (107 ± 10.6%) between Marie Louise and Hatherly for the leachate; and for sediment hexane still had the highest recovery rates ranging from dTBP (95.9 ± 9.64%) to dTBP (117 ± 10.7%) between Marie Louise and Hatherly. Hexane was used as the extracting solvent for the leachate and sediment from here onwards.

Ten millitres of the selected DES for the study was used to extract 5 mL of the leachate samples (see Supplementary Fig S1(b)). The highest recovery rates of IS in all the samples were obtained using DES-3 as the solvent of extraction ranging from dTPrP (68.6 ± 1.23%) in Hatherly to dTBP (113 ± 5.02%) in Marie Louise.

Due to the viscous nature of DES-4, DES-2 and DES-6, water was added at different fractions to dilute and reduce the viscosities of the DES in order to extract OPFRs in sediment. Good IS recoveries were obtained with DES-4(b) (2.50 mL water added), DES-2(b) (1.25 mL water added) and DES-6(d) (3.75 mL water added) in Hatherly. In Marie Louise, good IS recoveries were obtained with DES-4(b) (1.25 mL water added), DES-2(d) (3.75 mL water added) and DES-6(a) (0.00 mL water added). Thereafter, the latter which gave good recoveries, were used as the extracting solvent for each DES group in Hatherly and Marie Louise sediment samples. A sample size of 0.75 g was extracted with only the best DES for each diluted viscous group DES-4, DES-2 and DES-6 in comparison to the recoveries obtained using the less viscous DES-1, DES-3, DES-5 in the samples, see Supplementary Fig S2(b). Hatherly and Marie Louise samples had the highest IS recoveries ranging from dTBP (102 ± 9.34%) to ^13^C_18_MTPP (108 ± 6.83%) for all compounds when using DES-1 to extract the sediment. Hence, DES-3 and DES-1 was used as the extraction solvents for the leachate and sediment thereafter.

Effect of sonication time: Sonication time for extraction of the leachate was optimised using hexane and DES-3 (see Supplementary Fig S3(c) and Fig S1(c)). The highest IS recoveries were obtained at 20 min sonication time from dTPP (88.0 ± 8.02%) in Marie Louise to dTBP (108 ± 8.80%) in Hatherly with hexane. Optimum sonication time using DES-3 was 5 min. The recoveries ranged from dTPrP (76.9 ± 2.53%) to dTPP (119 ± 3.10%) in Marie Louise and dTBP (98.3 ± 9.97%)–^13^C^18^MTPP (107 ± 3.03%) in Hatherly. The optimum sonication time was 20 min (hexane) and 5 min (DES-3) for the leachate.

Supplementary Fig S4(c) and Fig S2(c) shows the sonication time that sediment samples were also optimised. Recovery rates for the spiked IS were the highest at 10 min ranging from dTBP (86.1 ± 8.01%) in Marie Louise to dTBP (103 ± 10.1%) in Hatherly using hexane; and using DES-1 IS recoveries were the highest at 10 min from Hatherly to Marie Louise ranging from dTPrP (40.2 ± 5.41%) to dTPP (115 ± 5.92%). Ten minutes was applied with organic solvents and DES in sediment onwards.

Effect of vortex time: Optimum vortex time for the leachate samples according to their IS recovery rates is 60 s, these ranged from Hatherly at ^13^C_18_MTPP (82.2 ± 8.26%) to Marie Louise at dTBP (104 ± 6.66%) using hexane (see Supplementary Fig S3(d)). Correspondingly using DES-3, the optimum vortex time was 60 s with recoveries ranging from Marie Louise at ^13^C_18_MTPP (57.6 ± 3.32%) to Hatherly at dTPP (111 ± 4.60%) (see Supplementary Fig S1(d)).

For sediment samples, the recoveries ranged from dTPrP (77.2 ± 843%) to ^13^C_18_MTPP (117 ± 3.24%) in Marie Louise and from ^13^C_18_MTPP (83.9 ± 5.55%) to dTPrP (98.1 ± 4.70%) in Hatherly at 60 s using hexane (see Supplementary Fig S1(d)). Using DES-1, all compounds in the Hatherly and Marie Louise samples showed low recoveries at vortex times 30 s and 120 s; high recoveries were observed at 60 s. The recoveries obtained at 60 s ranged between ^13^C_18_MTPP (57.6 ± 4.02%) and dTPP (111 ± 5.61%) for sediment (see Supplementary Fig S1(d)). The optimum vortex time used for applications in the leachate and sediment using organic and DES is 60 s.

Effect of centrifuge speed: In the leachate, centrifuge speed 3500 RPM gave the highest IS recoveries in Hatherly and Marie Louise ranging from ^13^C_18_MTPP (85.2 ± 7.32%) to dTBP (113 ± 3.34%) and dTPP (93.1 ± 7.35%)–dTBP (109 ± 9.56%) using hexane (see Supplementary Fig S3(e)). In the Marie Louise and Hatherly samples, the highest recoveries were obtained at 3500 RPM, the IS recovery rates ranged from ^13^C_18_MTPP (83.8 ± 2.62%) to dTPrP (98.6 ± 3.30%) using DES-3 (see Supplementary Fig S1(e)). Centrifuge speed 3500 RPM was optimum with both organic and DES in the leachate.

In sediment see Supplementary Fig S4(e) and Fig S2(e), high recoveries in Hathely and Marie Louise ranging from ^13^C_18_MTPP (81.2 ± 7.38%) to dTPrP (121 ± 10.2%) and dTPP (82.9 ± 6.78%)–dTBP (98.8 ± 6.78%) for all the compounds were obtained at 4000 RPM with hexane; and with DES-1 high recoveries in Marie Louise and Hatherly ranging from dTPP (95.3 ± 4.03%) to ^13^C_18_MTPP (99.9 ± 7.80%) for the spiking IS compounds were obtained at 2500 RPM. The optimum centrifuge speed applied moving forward was 4000 RPM and 2500 RPM for hexane and DES, respectively.

Effect of centrifuge time: Internal standard (IS) recoveries of spiked leachate were the highest at 20 min centrifuge times between dTBP (97.1 ± 6.75%) in Hatherly to dTBP (109 ± 6.96%) in Marie Louise using hexane (see Supplementary Fig S3(f)). The recoveries were high at 5 min centrifuge time ranging between dTBP (83.1 ± 8.79%) and dTPrP (113 ± 6.57%) (Marie Louise–Hatherly) using DES. Therefore, 20 min and 5 min centrifuge speeds were applied in the leachate samples using hexane and DES-3, respectively (see Supplementary Fig S1(f)).

Using hexane (see Supplementary Fig S4(f)) in sediment, high spiking IS recovery rates were obtained at 5 min at Marie Louise from ^13^C_18_MTPP (88.3 ± 10.53%) to dTPrP (113 ± 6.92%) and in Hatherly from dTBP (94.2 ± 5.17%) to dTPrP (110 ± 5.55%). With DES-1 (see Supplementary Fig S2(f)) 5 min gave high spiked IS recoveries ranging from ^13^C_18_MTPP (89.9 ± 6.22%) to dTBP (119 ± 4.05%) (Marie Louise–Hatherly). Subsequently, 5 min was used as the optimum centrifuge time for sediment extraction with both organic and DES.

DES and OPFR compatibility: The mechanism of interaction between the DES solvents and OPFRs that leads to the obtained optimal conditions of extraction, is not yet understood. However, the advantage of using DES is due to their physicochemical properties such as their thermal stability, tunable viscosities, and their broad polarity range. OPFRs have a wide range of polarities and solubilities, so understanding the selectivity that occurs between the compounds and DES will be an added advantage. A study by Shekaari et al.^32^ explored DES as a co-solvent by studying the solubility of acetaminophen in choline chloride with urea and oxalic acid as HBDs. Increased solubility with increasing DES concentrations and temperatures, especially when oxalic acid was used as an HBD was observed. Also, Cao et al.^33^ studied the reaction between DES and flavonoids, the results showed that the higher the choline chloride content (HBA) in the DES or DES solutions, the better the extraction efficiency and that the HBDs may react with the target compounds to affect the extraction efficiency. These studies show that the compounds of interest will either be compatible with the DES increase in HBA, HBD or the presence of both. In the case of this study, the interaction of each compound with DES will have to be investigated to gain understanding on which compounds better interact with which molecule of the DES during extraction.

### Method validation

The optimal LLE and SLE OPFR extraction conditions for the leachate and sediment using organic solvents and DES are presented in Table 2. Optimum solvents, hexane, and DES (DES-3 and DES-1) were applied to the optimum LLE and SLE conditions to validate the extraction procedure, by evaluating IS % recoveries obtained.

Tables 3 and 4 show the mean (n = 2) IS % recoveries obtained for QC blanks and real samples from the two selected landfill sites, using hexane and DES (DES-3 and DES-1) for extraction. As can be seen in Table 3, the percentage recoveries of real and blank leachate samples using hexane as the extracting solvent was 62.4 ± 1.02–98.7 ± 2.30% for de-ionised water (QC blank); 59.0 ± 7.54–93.4 ± 3.21% for Hatherly landfill site and 61.9 ± 2.41–98.0 ± 3.45% for Marie Louise landfill sites. Recoveries for sediment samples ranged from 63.6 ± 2.16 to 100 ± 4.21% (sodium sulphate (Na_2_SO_4_) QC blank); 59.9 ± 3.85–94.9 ± 4.53% (Hatherly landfill site) and 62.5 ± 5.34–98.9 ± 4.53% (Marie Louise landfill site).

**Table 3:** Mean (n = 2) spiked surrogate percentage recoveries ± standard deviations (SD) of real and blank leachate and sediment samples using hexane for extraction.

	dTPP (IS)	dTBP (IS)	dTPrP (IS)	^13^C_18_MTPP (IS)
**Leachate (Hex)**				
QC blank	73.3 ± 3.03	98.7 ± 2.30	85.3 ± 2.7	62.4 ± 1.02
Hatherly	69.3 ± 4.21	93.4 ± 3.21	80.7 ± 5.01	59.0 ± 7.54
Marie Louise	72.7 ± 3.41	98.0 ± 3.45	84.6 ± 9.71	61.9 ± 2.41
**Sediment (Hex)**				
QC blank	74.6 ± 5.5	100 ± 4.21	86.9 ± 2.02	63.6 ± 2.16
Hatherly	70.4 ± 4.82	94.9 ± 2.56	81.9 ± 3.32	59.9 ± 3.85
Marie Louise	73.4 ± 3.21	98.9 ± 4.53	85.4 ± 2.06	62.5 ± 5.34

**Table 4:** Mean (n = 2) spiked surrogate percentage recoveries of real and blank leachate and sediment samples extracted using DES-1 and choline DES-3 for extraction.

	dTPP (IS)	dTBP (IS)	dTPrP (IS)	^13^C_18_MTPP (IS)
**Leachate (DES-3)**				
QC blank	97.2 ± 3.21	101 ± 4.65	89.0 ± 0.08	89.1 ± 3.03
Hatherly	83.5 ± 6.89	99.0 ± 5.02	95.5 ± 8.56	62.6 ± 7.56
Marie Louise	83.7 ± 5.23	109 ± 6.19	85.8 ± 5.32	72.8 ± 9.18
**Sediment (DES-1)**				
QC blank	94.3 ± 1.87	120 ± 2.35	104 ± 2.23	95.2 ± 2.62
Hatherly	80.4 ± 4.56	105 ± 4.42	91.9 ± 6.13	59.9 ± 7.41
Marie Louise	77.0 ± 2.35	103 ± 5.32	89.7 ± 4.53	75.6 ± 9.97

In Table 4, the percentage recoveries for the leachate using DES-3 ranged from 89.0 ± 0.08 to 101 ± 4.56% (de-ionised water (QC blank)); 62.6 ± 7.56–99.0 ± 5.02% (Hatherly landfill site) and 72.8 ± 9.18–109 ± 6.19% (Marie Louise landfill site). These percentage recoveries are higher than those obtained using hexane (Table 3) as the extracting solvent. Sediment recoveries using DES-1 ranged from 94.3 ± 1.87 to 120 ± 2.35% (Na_2_SO_4_ QC blank); 59.9 ± 7.41–105 ± 4.42% (Hatherly landfill site) and 75.6 ± 9.97–103 ± 5.32% (Marie Louise landfill site). Correspondingly, the values are significantly higher than those exhibited by hexane in Table 3.

CRM (EDF-2525 contaminated natural matrix reference material) was subjected to SLE duplicated extractions using hexane and DES-1 (see Supplementary Table S4 and Table S5). CRM recoveries obtained using hexane ranged from 81.2 to 109% and with DES-1 85.2–118%. DES-1 had higher OPFR recoveries compared to hexane. The percentage recoveries observed in the present study are comparable to the values reported in the literature (see Supplementary Text S6) for the extraction of OPFRs in aqueous and soil media. Hence, due to the comparable extraction performance and, in other instances, outstanding performances of green DES to extract OPFRs in landfill leachate and sediment samples using LLE and SLE, DES can serve as good replacements for organic solvents used to extract OPFRs in landfill leachates and sediments.

### Solvent application and OPFR quantification of landfill leachate and sediment

Leachate OPFR concentrations: The mean (n = 2) concentrations of targeted OPFRs in the leachate from Tshwane and Johannesburg landfill sites are tabulated for each site in Table 5. All concentrations reported in this study are above LOQs in their respective media. Using hexane for extraction, 11 (80%) OPFRs (TPP, TMTP, TCEP, TCPP, T21PPP, TBEP, TEHP, TPrP, TEP, and TDCPP) were detected in landfill leachate samples. TCEP was found in all the selected landfill leachate samples. TOTP, T35DMPP, and HEDP were not detected in any of the leachate samples in the selected landfill sites. TDCPP and TEP exhibited the highest concentrations of 516 ± 8.10 ng/L and 465 ± 3.50 ng/L in the Onderstepoort landfill site. Also, Onderstepoort exhibited the highest sum of organophosphorus flame retardants (ƩOPFRs) in the leachate at 981 ng/L.

**Table 5:** Mean (n = 2) OPFR concentrations ± SD in the leachate (ng/L) from eight selected landfill sites extracted using hexane and DES-3.

LOQs	TOTP	TPP	TMTP	TCEP	TCPP	TPTP	T35DMPP	T21PPP	TBEP	TEHP	EHDP	TPrP	TEP	TDCPP	ƩOPFRs
	0.01	0.01	0.01	0.01	0.01	0.01	0.001	0.01	0.01	0.01	0.01	0.001	0.01	0.01	
**Hexane (ng/L)**															
Hat	< LOQ	< LOQ	7.05 ± 2.02	0.735 ± 0.133	0.794 ± 0.021	10.6 ± 2.32	< LOQ	< LOQ	61.6 ± 3.45	0.538 ± 0.156	< LOQ	< LOQ	< LOQ	< LOQ	81.3
Sosh	< LOQ	1.03 ± 0.346	14.9 ± 3.00	1.49 ± 0.056	5.36 ± 1.08	9.75 ± 3.80	< LOQ	0.739 ± 0.002	139 ± 2.02	< LOQ	< LOQ	< LOQ	< LOQ	< LOQ	172
Ond	< LOQ	< LOQ	< LOQ	0.695 ± 0.190	< LOQ	< LOQ	< LOQ	< LOQ	< LOQ	< LOQ	< LOQ	< LOQ	465 ± 3.50	516 ± 8.10	981
Gar	< LOQ	< LOQ	< LOQ	0.633 ± 0.311	< LOQ	< LOQ	< LOQ	< LOQ	< LOQ	< LOQ	< LOQ	5.27 ± 1.02	< LOQ	< LOQ	5.90
Enn	< LOQ	0.493 ± 0.061	< LOQ	1.05 ± 0.013	4.35 ± 2.02	< LOQ	< LOQ	< LOQ	8.34 ± 3.56	< LOQ	< LOQ	0.002 ± 0.001	90.2 ± 4.42	62.7 ± 6.71	167
Gou	< LOQ	< LOQ	7.55 ± 1.035	1.32 ± 0.234	< LOQ	7.40 ± 2.40	< LOQ	1.31 ± 0.051	105 ± 5.10	1.26 ± 0.650	< LOQ	< LOQ	< LOQ	< LOQ	123
Mar	< LOQ	0.291 ± 0.004	< LOQ	0.717 ± 0.350	1.64 ± 0.324	< LOQ	< LOQ	< LOQ	13.8 ± 3.40	< LOQ	< LOQ	0.177 ± 0.021	148 ± 5.30	140 ± 5.32	304
Rob	< LOQ	0.106 ± 0.084	17.2 ± 1.56	0.796 ± 0.040	5.59 ± 0.892	20.5 ± 3.40	< LOQ	0.891 ± 0.310	124 ± 2.06	< LOQ	< LOQ	< LOQ	68.4 ± 2.62	76.8 ± 6.89	314

LOQ	1.00	0.01	0.01	0.10	0.10	1.00	0.01	0.01	2.00	3.00	2.00	0.01	3.00	5.00	
**DES-3 (ng/L)**															
Hat	35.0 ± 4.85	1.94 ± 0.970	33.2 ± 8.90	3.04 ± 0.10	0.787 ± 0.05	33.6 ± 6.20	36.3 ± 8.56	1.37 ± 0.20	154 ± 2.50	169 ± 8.59	14.3 ± 5.01	8.92 ± 2.32	152 ± 2.99	110 ± 6.71	755
Sosh	37.0 ± 0.530	0.42 ± 0.230	71.9 ± 1.23	1.61 ± 0.89	19.3 ± 2.58	164 ± 2.08	12.7 ± 3.52	13.5 ± 3.03	156 ± 3.10	210 ± 2.53	226 ± 4.60	206 ± 2.15	134 ± 5.30	177 ± 7.63	1433
Ond	26.4 ± 3.45	3.86 ± 1.02	8.31 ± 2.56	2.25 ± 1.42	18.6 ± 5.02	29.2 ± 9.18	5.42 ± 1.97	< LOQ	145 ± 5.20	273 ± 3.88	85.2 ± 2.02	8.25 ± 3.50	86.6 ± 2.40	126 ± 4.23	819
Gar	80.9 ± 3.88	0.615 ± 0.050	91.7 ± 4.65	< LOQ	11.8 ± 3.60	82.5 ± 5.60	18.0 ± 3.53	< LOQ	133 ± 5.00	115 ± 32.1	310 ± 4.20	5.56 ± 2.50	72.9 ± 2.03	199 ± 3.67	1123
Enn	33.4 ± 0.856	2.22 ± 0.52	26.5 ± 6.19	0.724 ± 0.320	32.3 ± 11.9	66.2 ± 12.5	2.80 ± 0.240	< LOQ	453 ± 8.10	161 ± 15.60	113 ± 5.03	5.75 ± 2.34	180 ± 4.03	191 ± 5.32	1270
Gou	8.14 ± 1.85	1.62 ± 0.53	< LOQ	0.653 ± 0.350	14.0 ± 9.71	5.51 ± 1.50	1.62 ± 0.20	0.444 ± 0.050	45.3 ± 2.06	42.7 ± 5.60	13.5 ± 3.34	0.06 ± 0.01	201 ± 7.43	175 ± 5.12	510
Mar	28.7 ± 5.90	4.40 ± 1.50	20.6 ± 2.89	1.78 ± 0.650	26.2 ± 4.50	32.4 ± 5.10	6.89 ± 1.02	6.21 ± 3.40	124 ± 6.20	200 ± 6.80	118 ± 5.00	22.8 ± 8.56	155 ± 8.90	172 ± 9.30	921
Rob	11.7 ± 0.530	0.05 ± 0.02	1.06 ± 0.05	< LOQ	5.97 ± 2.50	3.25 ± 1.45	0.05 ± 0.02	0.527 ± 0.070	181 ± 9.32	59.9 ± 2.70	15.6 ± 3.80	2.11 ± 5.20	93.3 ± 9.80	206 ± 8.10	580

Ond = Onderstepoort, Gar = Ga-Rankuwa, Sosh = Soshanguve, Hat = Hatherly, Enn = Ennerdale, Rob = Robinson Deep, Mar = Marie Louise, n = number of sample replicates per landfill site.

Compared to the OPFR concentrations in the leachate that were extracted using hexane, with DES-3, 14 (100%) OPFRs ( TOTP, TPP, TCPP, TPTP, T35DMPP, TBEP, TEHP, EHDP, TPrP, TEP, and TDCPP) were extracted in all the selected landfill sites (Table 5). TMTP, TCEP, and T21PPP were < LOQ in the Goudkoppies, Ga-Rankuwa, Robinson Deep, Onderstepoort, and Ennerdale landfill sites. TBEP showed the highest concentration of 453 ± 8.10 ng/L in the Ennerdale landfill site. The highest ƩOPFRs was exhibited in the Soshanguve landfill site at 1433 ng/L.

The extractability of compounds like TOTP, T35DMPP, and EHDP using DES-3 that were < LOQ with hexane can be attributed to the polarizability (0.527) effect of DES-3, which may have enhanced the extraction of OPFRs^34,35^. A high ƩOPFRs was observed in the Onderstepoort leachate using hexane, and a similar observation was made with Soshanguve leachate using DES-3 for extraction. These could be associated with the municipal waste received by the City of Tshwane (199 tonnages) in June 2019, which was higher than the waste received by the City of Johannesburg landfill sites (89 tonnages) (see Supplementary Fig S8). It is, therefore, possible that the observed high levels of OPFRs in the landfill sites may be due to OPFR-treated food contact items, packaging, canning and others, dumped in the landfills^36^.

TPP was < LOQ using hexane in Hatherly, Onderstepoort, Ga-Rankuwa and Goudkoppies, but using DES-3, TPP concentrations ranged from 0.05 ± 0.02 ng/L (Robinson Deep)–3.86 ± 1.02 ng/L (Onderstepoort). TMTP was < LOQ in Onderstepoort, Ga-Rankuwa and Ennerdale with hexane. However, it was detected at 8.31 ± 2.56 ng/L, 91.7 ± 4.65 ng/L and 26.5 ± 0.52 ng/L in the same sites using DES-3. Also, T21PPP was detected < LOQ in Hatherly and Marie Louise with hexane, but it was detected in the same leachates using DES-3 at 1.37 ± 0.20 ng/L and 6.21 ± 3.40 ng/L. The low-volatility and high thermal stability of DES may have played a significant role in ensuring that all extracted compounds remain in the DES^37^. This data shows that extractability of OPFRs using DES is higher compared to hexane.

The OPFR leachate concentrations obtained using hexane and DES were lower than the results of the study by Shahbodaghi et al.^23^, where the highest concentration was 3920 ng/L for TCPP in wastewater and the lowest was 150 ng/L for TPrP in well water using BTPPB: 1-dodecanol DES. Apart from the DES used, the high concentrations could be due to the sample matrix used, and the factors contributing to the OPFR contaminant levels. The compound with the highest concentration using hexane in this study was TDCPP at 516 ± 8.10 ng/L; whereas with DES-3 it was TBEP at 453 ± 8.10 ng/L. The lowest concentration using hexane was TPrP at 0.002 ± 0.001 ng/L and 0.05 ± 0.02 ng/L for TPP and T35DMPP using DES-3. These high TDCPP and TBEP concentrations could be due to leaching from OPFRs-containing wastes disposed into the landfill sites. Products such as nursing pillows, car seats and polyurethane foam from furniture are known to be treated with OPFRs^38^. At the end of their life-cycle, most of these products are disposed of in landfill sites with little or no recycling or treatment.

Sediment OPFR concentrations: The mean (n = 2) concentrations of OPFRs extracted using hexane and DES-1 for sediment are shown in Table 6. The OPFRs, TOTP, TPP, TCEP, TPTP, T35DMPP, T21PPP and THEP were extracted in some landfill sites with hexane, but TMTP, TCPP, TBEP, EHDP, TPrP, TEP and TDCPP were < LOQ. When using organic solvents like hexane that are volatile, volatile OPFRs with high vapour pressures may escape, resulting in their concentrations being < LOQ^39^. Using hexane, the dominant OPFR was TPTP at 135 ± 2.89 ng/g dw and 133 ± 1.23 ng/g dw in Hatherly and Ga-Rankuwa. Ga-Rankuwa also showed the highest ƩOPFRs in sediment at 298 ng/g dw.

**Table 6:** Mean (n = 2) OPFR concentrations ± SD in sediment (ng/g dw) from eight selected landfill sites extracted using hexane and DES-1.

LOQ	TOTP	TPP	TMTP	TCEP	TCPP	TPTP	T35DMPP	T21PPP	TBEP	TEHP	EHDP	TPrP	TEP	TDCPP	ƩOPFRs
	0.08	0.05	0.12	0.10	0.10	0.12	0.12	0.08	0.10	0.10	0.05	0.10	0.05	0.12	
**Hexane (ng/g dw)**															
Hat	63.6 ± 0.530	< LOQ	< LOQ	< LOQ	< LOQ	135 ± 2.89	< LOQ	17.2 ± 4.50	< LOQ	< LOQ	< LOQ	< LOQ	< LOQ	< LOQ	216
Sosh	< LOQ	4.37 ± 0.890	< LOQ	< LOQ	< LOQ	< LOQ	< LOQ	< LOQ	< LOQ	< LOQ	< LOQ	< LOQ	< LOQ	< LOQ	4.37
Ond	< LOQ	< LOQ	< LOQ	3.73 ± 0.150	< LOQ	< LOQ	< LOQ	< LOQ	< LOQ	< LOQ	< LOQ	< LOQ	< LOQ	< LOQ	3.74
Gar	66.6 ± 1.234	< LOQ	< LOQ	< LOQ	< LOQ	133 ± 1.23	49.7 ± 4.32	17.8 ± 4.42	< LOQ	30.3 ± 2.03	< LOQ	< LOQ	< LOQ	< LOQ	298
Enn	< LOQ	< LOQ	< LOQ	< LOQ	< LOQ	< LOQ	< LOQ	< LOQ	< LOQ	< LOQ	< LOQ	< LOQ	< LOQ	< LOQ	64.9
Gou	< LOQ	< LOQ	< LOQ	< LOQ	< LOQ	< LOQ	< LOQ	< LOQ	< LOQ	< LOQ	< LOQ	< LOQ	< LOQ	< LOQ	< LOQ
Mar	< LOQ	2.48 ± 0.032	< LOQ	7.65 ± 3.55	< LOQ	< LOQ	< LOQ	1.96 ± 0.62	< LOQ	< LOQ	< LOQ	< LOQ	< LOQ	< LOQ	12.1
Rob	< LOQ	< LOQ	< LOQ	< LOQ	< LOQ	< LOQ	< LOQ	< LOQ	< LOQ	< LOQ	< LOQ	< LOQ	< LOQ	< LOQ	< LOQ

LOQ	0.10	10.0	1.00	10.0	5.00	1.00	0.10	1.00	10.0	0.10	5.00	5.00	10.0	0.10	
**DES-1 (ng/g dw)**															
Hat	< LOQ	50.1 ± 4.18	3.59 ± 2.93	144 ± 1.52	192 ± 2.76	17.3 ± 9.22	3.37 ± 1.75	24.1 ± 4.47	25.5 ± 20.8	7.64 ± 3.23	17.6 ± 0.76	240 ± 3.61	< LOQ	< LOQ	725
Sosh	24.6 ± 20.0	30.1 ± 2.02	24.7 ± 20.1	175 ± 47.7	134 ± 35.5	33.8 ± 27.5	13.6 ± 1.01	48.0 ± 3.22	< LOQ	11.4 ± 6.83	31.7 ± 14.7	308 ± 11.2	< LOQ	< LOQ	834
Ond	< LOQ	105 ± 16.0	< LOQ	153 ± 7.05	146 ± 10.3	< LOQ	< LOQ	< LOQ	< LOQ	< LOQ	< LOQ	395 ± 2.24	< LOQ	< LOQ	799
Gar	0.16 ± 0.13	130 ± 67.6	8.93 ± 4.28	148 ± 25.6	147 ± 17.1	11.4 ± 1.96	7.92 ± 6.46	46.2 ± 13.4	38.5 ± 13.4	16.3 ± 8.95	9.32 ± 3.95	321 ± 6.55	< LOQ	< LOQ	884
Enn	17.9 ± 8.95	62.5 ± 9.50	119 ± 63.6	168 ± 51.8	132 ± 56.8	38.6 ± 23.3	22.7 ± 6.90	64.8 ± 32.4	109 ± 54.5	21.9 ± 10.9	50.6 ± 12.0	246 ± 15.0	171 ± 85.5	< LOQ	1224
Gou	24.1 ± 12.0	55.2 ± 18.3	< LOQ	108 ± 23.2	48 ± 24.0	32.5 ± 16.2	25.1 ± 6.00	44.2 ± 22.1	57.9 ± 28.9	20.7 ± 10.3	34.2 ± 18.7	191 ± 19.2	150 ± 75.0	< LOQ	790
Mar	43.1 ± 21.5	35.4 ± 4.85	2.62 ± 1.31	133 ± 44.3	124 ± 62.0	< LOQ	< LOQ	9.04 ± 4.52	< LOQ	12.5 ± 6.25	28.1 ± 3.06	281 ± 40.0	194 ± 97.0	< LOQ	862
Rob	6.61 ± 3.30	57.8 ± 14.0	< LOQ	121 ± 10.7	180 ± 81.7	19.8 ± 6.10	< LOQ	20.4 ± 10.2	66.6 ± 33.3	1.11 ± 0.55	2.60 ± 0.800	204 ± 1.02	122 ± 61.0	< LOQ	834

Ond = Onderstepoort, Gar = Ga-Rankuwa, Sosh = Soshanguve, Hat = Hatherly, Enn = Ennerdale, Rob = Robinson Deep, Mar = Marie Louise, n = number of sample replicates per landfill site.

Using DES-1 to extract OPFRs from landfill sediment, all targeted compounds apart from TDCPP, which was < LOQ, were quantified above LOQ. TPrP had the highest concentration of 395 ± 2.24 ng/g dw in Onderstepoort and 308 ± 11.2 ng/g dw in the Soshanguve landfill site. The landfill site that showed the highest ƩOPFRs in sediment was Goudkoppies at 1224 ng/g dw. Hexane and DES-1 exhibited extraction efficiencies in line with their perfomances during method development and validation, in that DES-1 extracted OPFRs in sediment better. The efficiency of DES-1 can be atributted to its physical property of being non-volatile and thermally stable, so that it retains all the targeted compounds.

Using hexane, T35DMPP and TEHP were < LOQ in all the landfill sites except in Ga-Rankuwa where they were detected at 49.7 ± 4.32 ng/g dw and 30.3 ± 2.03 ng/g dw. Correspondingly, using DES-1, T35DMPP concentrations were < LOQ in Onderstepoort, Marie Louise and Robinson Deep; and TEHP was < LOQ only in Onderstepoort. In the sites where OPFRs were detected < LOQ by both the organic and DES extracting solvents, it is possible that the compounds may be present at very low levels that could not be detected. In the landfill sites where OPFRs were detected using DES-1, T35DMPP ranged from 3.37 ± 1.75 ng/g dw (Soshanguve) to 25.1 ± 6.00 ng/g dw (Goudkoppies); and TEHP from 1.11 ± 0.55 ng/g dw (Robinson Deep) to 20.7 ± 10.3 ng/g dw (Goudkoppies) in Johannesburg and from 7.64 ± 3.23 ng/g dw (Hatherly) to 16.3 ± 8.95 ng/g dw (Ga-Rankuwa) in Tshwane landfill sites. Higher OPFRs sediment concentrations when using DES-1 were observed in the Johannesburg landfill sites, and this observation can be attributed to the adsorption of OPFRs in the sediment in the leachate pond. It is a well known fact that sediment serves as a sink for most pollutants. The geomembrane liners in Johannesburg landfill sites may have played a role in ensuring that compounds are contained in the leachate pond, hence the higher concentrations observed in Johannesburg landfill sites^40^.

Supplementary Tables S5 shows the comparison of OPFR concentrations in landfill leachate and sediment from studies around the world and the present study. In the present study, the ∑OPFR in the leachate extracted with hexane ranged from 5.90 ng/L (Ga-Rankuwa) to 981 ng/L (Onderstepoort); and using DES-3 the ∑OPFR ranged from 510 ng/L (Goudkoppies) to 1433 ng/L (Soshanguve). Concentrations in this study were higher than in most studies reporting OPFRs in the leachate, except the study by Sibiya et al.^15^ (556–17,200 ng/L) in South Africa and Yasuhara^41^ (4.1 − 5430 ng/L) in Japan, which reported concentrations that were higher than those reported in the current study.

The ∑OPFR concentrations in sediment from the present study using hexane were < LOQ—298 ng/g dw, and when using DES-1 it was 725–1224 ng/g dw. Hexane-extracted OPFRs were lower than those reported in landfill sediment studies around the world (see Supplementary Table S6). When using DES-1, the concentrations were higher than in the study by Wang et al.^17^ (< MDL–548) in China and Sibiya et al.^15^ (< LOQ—741) in South Africa.

Overall, more OPFR compounds were extracted in the leachate using DES, and a similar observation was made when sediment was extracted. This data demonstrates the high extractability of OPFRs with green DES, which is biodegradable in the environment and non-toxic to humans compared to harzadous hexane^42^.

## Conclusions

Organic solvents are volatile and have physical properties (boiling point, solubility, polarity, etc.) that lead to toxicity to the user, society, and the environment. Hence, in this study, the extractability of OPFRs in the leachate and sediment using organic solvents in comparison to green choline based DES was assessed. It was found that hexane extracted OPFRs in the leachate (59.0–98.7%) and sediment (59.9–100%) better in comparion to other organic solvents that were used neat and as mixtures. However, the extractability of OPFRs using DES-3 in the leachate (62.6–109%) and DES-1 in sediment (59.9–120%) was on average 10% better than hexane. A similar trend was observed in OPFRs concetrations from landfill leachate and sediment samples when hexane and DES were applied to extract OPFRs. The ability of DES-3 and DES-1 to extract TOTP, T35DMPP and EHDP, compared to non-extractability of these compounds using hexane, attests to the better extractability of OPFRs using DES over hexane. This capability was attributed to the non-volatility and thermal stability of choline-based DES in comparison to hexane, which is volatile, and this characteristic can lead to possible losses of analytes during extraction. Thus, green DES can be used with confidence as suitable replacements for organic solvents such as hexane in extracting OPFRs in landfill leachates and sediment.

## Supplementary Information

Supplementary Information.

## Data Availability

All data generated or analysed during this study are included in this published article [and its supplementary information files].
