# Supplementary material for: Comparison of the extractability of organophosphorus flame retardants in landfill media using organic and green solvents
Source: Sci Rep. 2022 Jun 9;12:9529. doi: 10.1038/s41598-022-13704-1 (PMC9184578; doi:10.1038/s41598-022-13704-1)
Supplement: Supplementary file 1 — Supplementary Information. [file 41598_2022_13704_MOESM1_ESM.docx]

# **SUPPLEMENTARY MATERIAL (SM)**

**Comparison of the extractability of organophosphorus flame retardants in landfill media using organic and green solvents**

Innocentia Velaphi Sibiya^1^, Okechukwu Jonathan Okonkwo^1^*

^1^Department of Environmental, Water and Earth Sciences, Tshwane University of Technology, Tshwane, South Africa

*Corresponding author: [OkonkwoOJ@tut.ac.za](mailto:OkonkwoOJ@tut.ac.za)

**Text S1**

Chemicals and materials

The following stable isotope labelled (SIL) standards were used in the present study: Triphenyl phosphate-d_15_ (dTPP), ^13^C_18_-Triphenyl phosphate (MTPP), Triethyl phosphate-d_15_ (dTEP), Tributyl phosphate-d_27_ (dTBP), Tri-n-propyl phosphate-d_21_ (dTPrP) and Tris (2-butoxy-(^13^C_12_) ethyl) phosphate (M6TBEP). Triphenyl phosphate (TPP), Tri-o-toly phosphate (TOTP), Tri-m-toly phosphate (TMTP), Tri-p-toly phosphate (TPTP), Tris (3,5-dimethylphenyl) phosphate (T35DMPP), Tris (2-isopropylphenyl) phosphate (T21PPP), Triethyl phosphate (TEP), Tri-n-propyl phosphate (TPrP), Tris (2-butoxyethyl) phosphate (TBEP) and Tris (2,3-dibromopropyl) phosphate (TDBPP); 2-Ethylhexyl diphenyl phosphate (EHDP), Tris (2-ethylhexyl) phosphate (TEHP), Tris (2-chloroethyl) phosphate (TCEP), Tris [(2R)-1-chloro-2-propyl] phosphate (TCPP), Tris (1,3-dichloro-2-propyl) phosphate (TDCPP) and Tributyl phosphate (TBP) used were all purchased from Wellington Laboratories (Guelph, Ontario, Canada). The sediment CRM used was EDF-2525 contaminated natural matrix reference material from the first worldwide interlaboratory study ^1^. Greiner Bio-one (Belgium) supplied polypropylene-PP tubes (50 mL). The 2 µm and 0.45 µm filters (Eppendorf) were supplied by VWR (Leuven, Belgium). All solvents (hexane, dichloromethane, ethyl acetate, acetone, methanol and formic acid) were HPLC grade and purchased from Acros Organics (Belgium). All solvents were tested for the investigated analytes contamination before use. Florisil cartridges (500 mg, 6 mL) were purchased from Supelco (Bellefonte, PA, USA). All glassware were washed with detergent and, thereafter, rinsed with Milli-Q water, acetone and finally oven dried. Prior to use, the clean glassware was rinsed with the extraction solvent. Ultrapure water was dispensed from Labostar ultrapure water equipment (Siemens, Germany) supplied by Separations, South Africa. Choline chloride (C_5_H_14_ClNO, 99.0%), urea ((NH_2_)_2_CO, 98 %) and oxalic acid dihydrate (C_2_H_2_O_4_·2H_2_O, 98%), were purchased from Sigma-Aldrich (Chemie GmbH, Steinheim, Germany).

**Text S2**

LC-MS/MS for target screening of OPFRs

The quantitative analyses were performed with a liquid chromatography-tandem mass spectrometry technique using a Shimadzu LC-MS-8030 model (Shimadzu USA Manufacturing Inc.,Canby, OR, USA), which was equipped with an electrospray ionisation source (ESI). The chromatographic separation of organophosphorus flame retardants was achieved on an InertSustain C18 column (3 μm particle size, 2.1 × 150 mm) (GL Sciences, Tokyo, Japan). The column temperature was set to 40 °C. The mobile phase consisted of a mixture of 0.1% formic acid (FA) in water (solvent A) and MeOH (solvent B). Binary gradient elution of 0.30 mL/min with 80 % pump B flow was used. starting with a mixture of 50% solvent A for 0.01 min; then from 50% to 20% (solvent A) for 3 min; 12 min, 20%; 15 min, 20%; 17 min, 50%; and 20 min, 50%. A sample aliquot of 10 μL was injected throughout the analysis. Standards and the test samples were subjected to a run-time of 20 min. All the target compounds in this study were analysed in negative ESI mode. The source heating-block was maintained at 400 °C, while the desolvation temperature of 250 °C was employed and a drying gas flow of 15 L/min. Nitrogen was used as the drying and nebulising gas (3 L/min), while the collision-induced dissociation (CID) gas was argon and was maintained at 230 kPa. The resulting fragment ions were monitored in multiple reaction monitoring (MRM) mode with a dwell time of 100 ms. During optimisation, a multiple reaction monitoring (MRM) scan mode was generated for all the studied compounds. In order to achieve maximum sensitivity, other conditions such as source temperature, capillary voltage, cone gas flows and desolvation temperatures were optimised. This was achieved by direct injection of 1 mg/L of each compound at a flow rate of 0.3 mL/min into the mass spectrometer. Upon the completion of these preliminary experiments, the precursor and product ions of each compound were identified as presented in supplementary Table S1 with their retention times. High correlation coefficients (R^2^) ranging from 0.997–0.999 were obtained from the LC-MS/MS for all targeted OPFR calibarations.

**Table S1** Retention times, precursor (Q) and product ions (q) of OPFRs using LC-MS/MS

| **Abbreviation​** | **RT​** | **Q​** | **q​** |
| --- | --- | --- | --- |
| TCEP​ | 7.52 | 284>222 | 284>99.0 |
| TOTP | 10.2 | 369>91.2 | 369>166 |
| TPP | 9.25 | 326>77.0 | 326>152 |
| TMTP | 10.2 | 369>91.1 | 369>243 |
| TCPP​ | 08.6 | 327>89.1 | 327>133 |
| TPTP | 10.2 | 369>165 | 369>91. |
| T35DMPP | 12.0 | 411>179 | 411>194 |
| T21PPP | 12.2 | 453>327 | 453>369 |
| TEHP | 12.1 | 435>99.1 | 435>113 |
| EHDP | 10.4 | 363>251 | 363>77.0 |
| TBEP​ | 09.7 | 399>199 | 399>101 |
| TPrP | 01.6 | 225>99.0 | 225>141 |
| TEP | 06.8 | 183>99.0 | 183>126 |
| TDCPP | 06.1 | 432>45.1 | 432>89.2 |
| **IS** | | | |
| dTEP | 7.08 | 197>134 | 197>166 |
| M6TBEP | 9.62 | 405>201 | 405>103 |
| dTCEP | 7.46 | 296>130 | 296>167 |
| **RS** | | | |
| dTPP | 9.19 | 342>82.1 | 342>88.2 |
| dTBP | 9.38 | 294>102 | 294>166 |
| dTPrP | 8.35 | 246>102 | 246>150 |
| ^13^C_18_MTPP | 5.73 | 344>327 | 344>89.1 |
| dTDCPP | 9.12 | 445>102 | 445>59.0 |

*IS=Internal standard (used for the instrument), RS=Recovery standard (for spiking)

**Text S3**

Synthesis of DESs

Choline chloride/Urea DES: Choline chloride (40 g) and urea (17.2 g) coded as DES-1 in this study was placed in a 500 mL beaker and heated to 80 °C, until the formation of a liquid substance. After 20 min, the homogenous colourless liquid was formed and, thereafter, used directly for the extraction of OPFRs from leachate and sediments samples.

Lewis acidic DES: Choline chloride (20 g) and zinc chloride (ZnCl_2_) (39 g) coded as DES-2 in this study was placed in a 500 mL beaker and heated to 110 °C, until the formation of a liquid substance. After 20 min, the homogenous colourless liquid formed was used directly for the extraction of OPFRs in leachate and sediment samples.

Bronsted acidic DES: Choline chloride/Oxalic acid dihydrate was prepared at molar ratios of 1:1 (20 g:18.04 g), 1:2 (20 g:54 g), and 2:1 (40 g:18 g), coded as DES-3, DES-4 and DES-5 were used as solvents for the extraction of the OPFRs from leachate and sediment samples. To prepare these mixtures, choline chloride was mixed with oxalic acid dihydrate in a beaker, which was heated to 90 °C, until the formation of a liquid substance. After 10 min, the homogenous colourless liquid formed was used directly for the extraction of OPFRs in leachate and sediment samples.

Carbohydrate-derived DES: Choline chloride (40 g) and D-Fructose (16 g) coded as DES-5 was placed in a beaker and heated to 180 °C, until the formation of a liquid substance. After 30 min, the homogenous colourless liquid formed was used directly for the extraction of OPFRs in leachate and sediment samples.

The more viscous DES like DES-4, DES-2 and DES-6, were further diluted with different fractions of water before extracting OPFRs from sediment as can be seen in Table 2.

**Table S2** Deep eutectic sovelvents and the volumes of water used for dilution for sediment extractions

| **DES name** | **Volume of DES in 5mL** |
| --- | --- |
| DES-2^(a)^ | 5.00 |
| DES-2^(b)^ | 3.75 |
| DES-2^(c)^ | 2.50 |
| DES-2^(d)^ | 1.25 |
| DES-4^(a)^ | 5.00 |
| DES-4^(b)^ | 3.75 |
| DES-4^(c)^ | 2.50 |
| DES-4^(d)^ | 3.75 |
| DES-6^(a)^ | 5.00 |
| DES-6^(b)^ | 3.75 |
| DES-6^(c)^ | 2.50 |
| DES-6^(d)^ | 1.25 |

*(a)=0.00 mL, (b)=1.25 mL, (c)=2.50 mL and (d)=3.75 mL water added to make 5 mL DES

**Table S3** DES charectarisation technique and instruments used

| **DES Characterisation technique** | **Instrument** |
| --- | --- |
| ^1^H NMR spectra | Varian NMR Gemini 400 spectrometer Varian 400 MHz |
| FTIR spectra | - Nicolet 380 FT-IR with a Smart Orbit diamond attenuated total reflectance cell - wave length resolution was set to 4 cm^−1^ - IR spectrum was collected in a range of 600 – 4000 cm^−1^ - Chemical structure and mass spectra were generated and processed by Chemograph Professional 6.5 Software. |


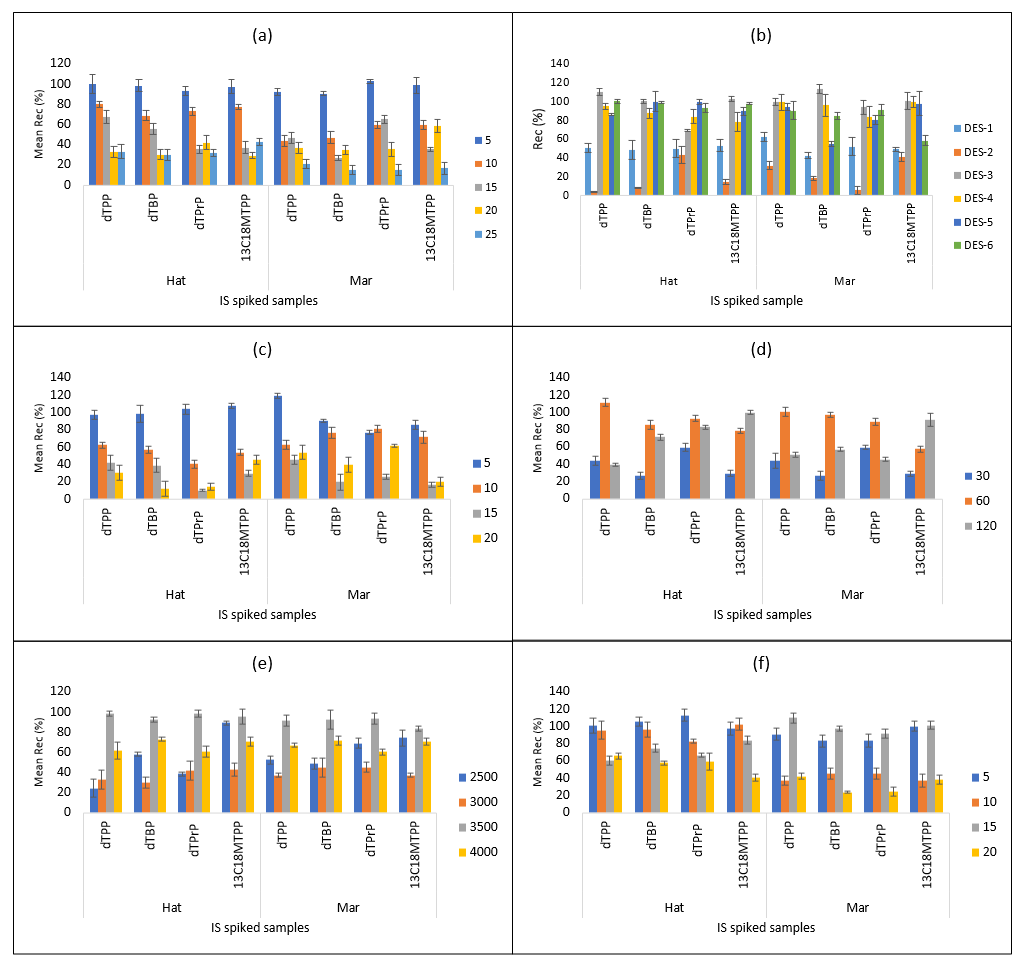


**Figure S1** Extraction efficiencies of parameters in leachate using green DES (a) = Sample volume (mL), (b)=extraction solvent, (c)=Sonication time (min), (d)=Vortex time (s), (e)=Centrifuge speed (RPM), (f)=Centrifuge time (min).


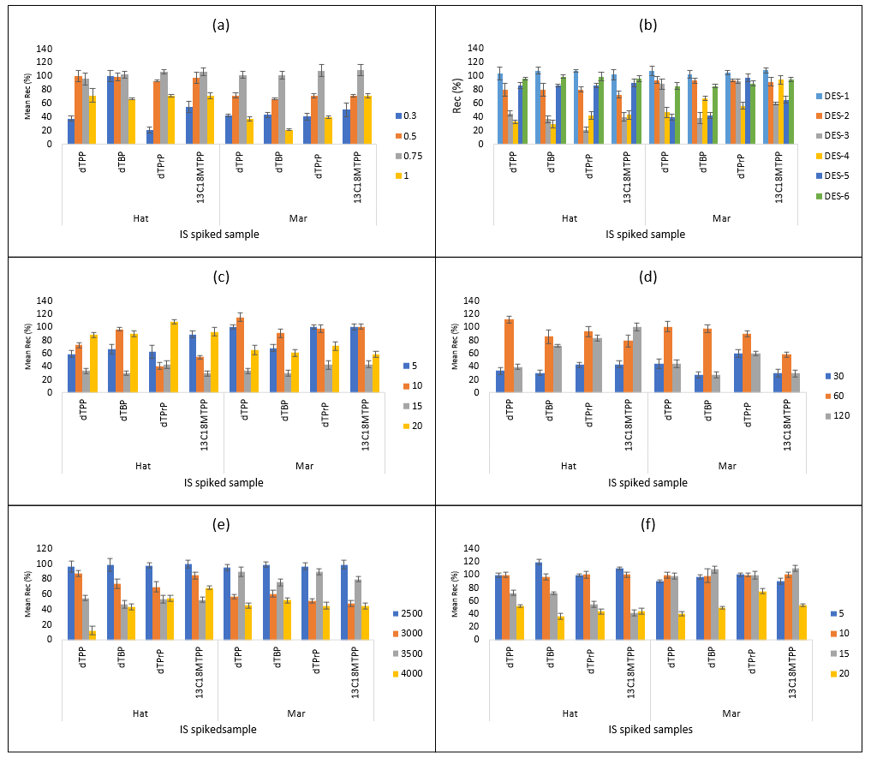


**Figure S2** Extraction efficiencies of parametersin sediment using green DES (a)=sample mass (g), (b)=extraction solvent, (c)=Sonication time (min), (d)=Vortex time (s), (e)=Centrifuge speed (RPM), (f)=Centrifuge time (min)


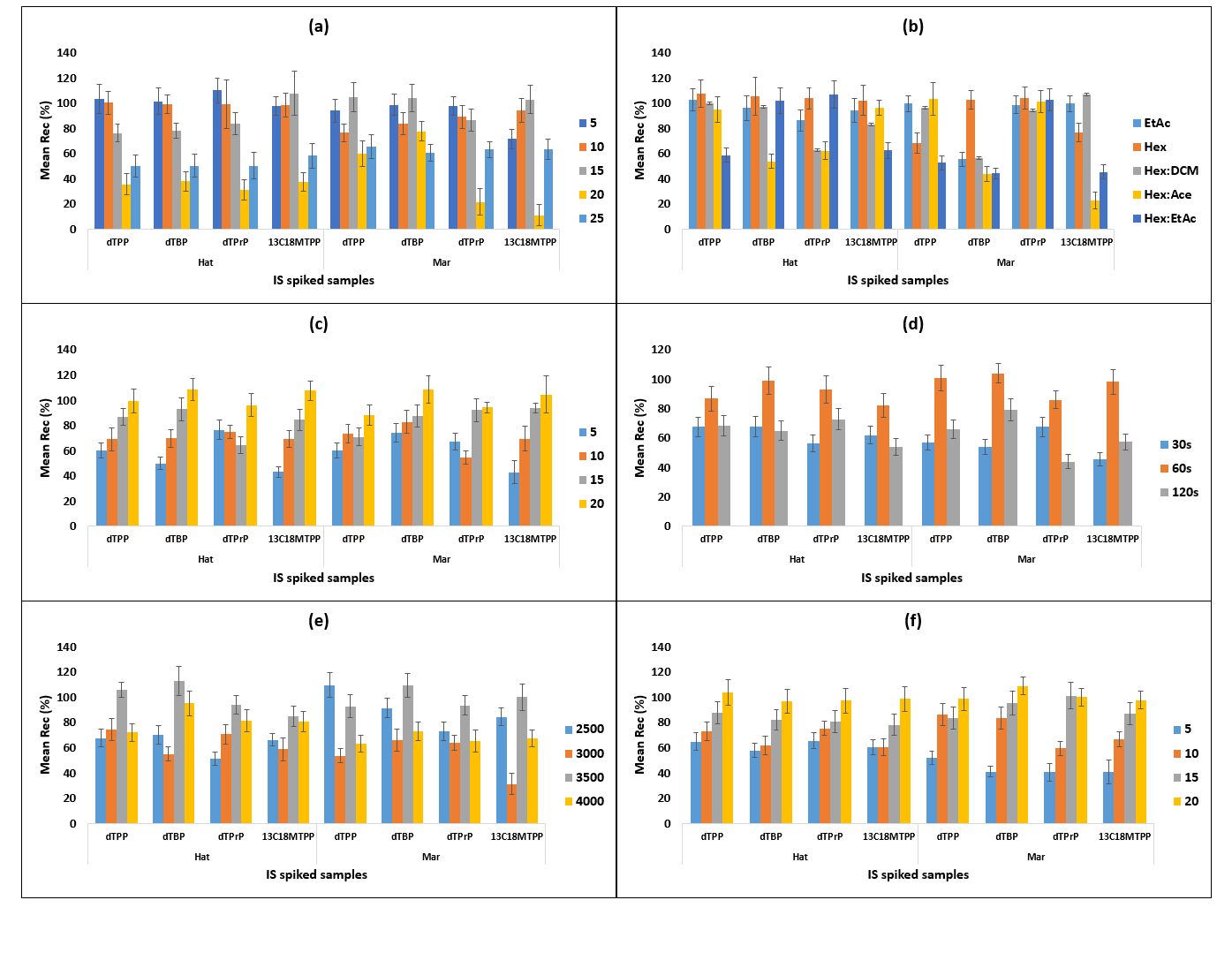


**Figure S3** Extraction efficiencies of parameters in leachate using organic solvents (a)=Sample volume (mL), (b)=extraction solvent, (c)=Sonication time (min), (d)=Vortex time (s), (e)=Centrifuge speed (RPM), (f)=Centrifuge time (min)


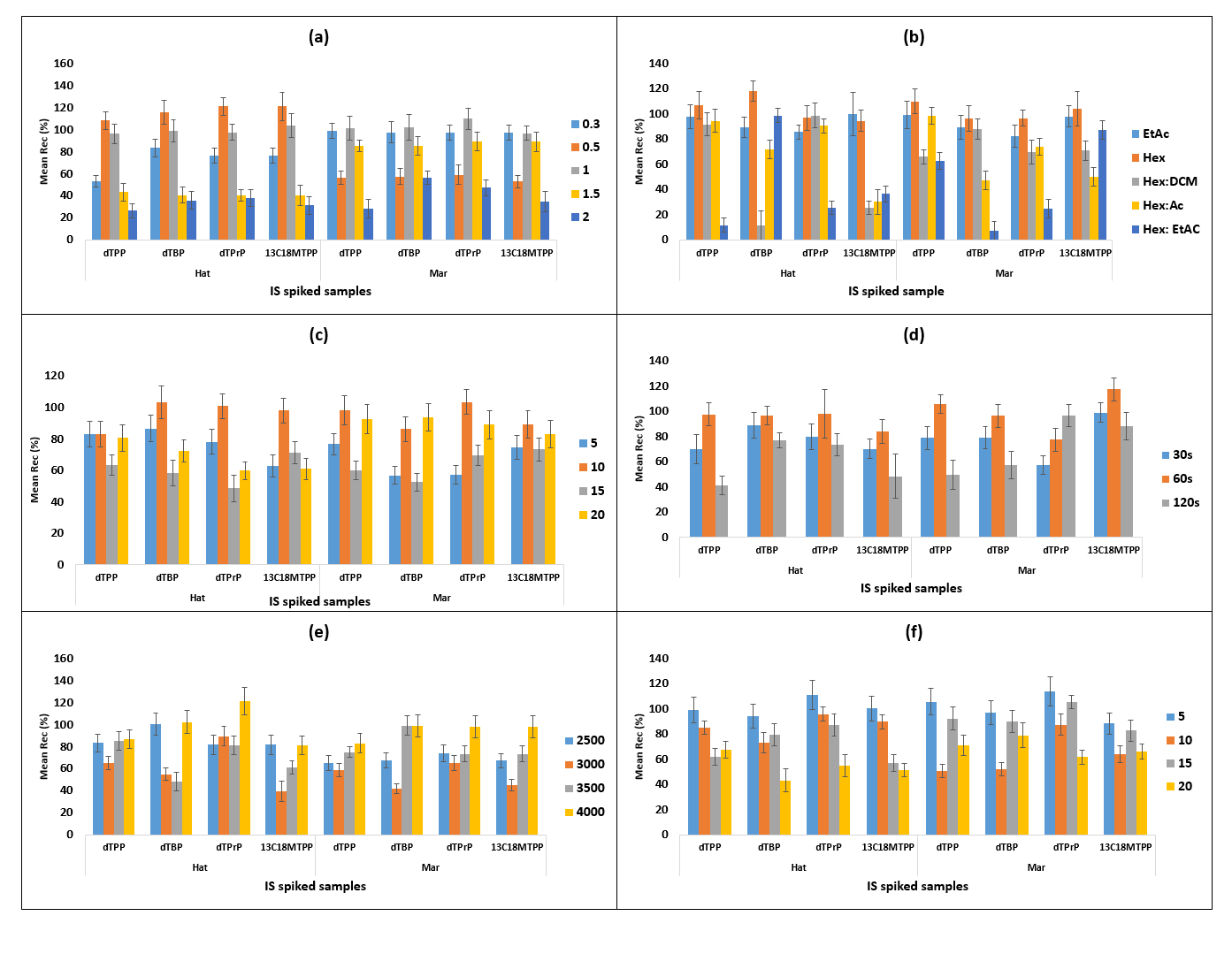


**Figure S4** Extraction efficiencies of parameters in sediment using organic solvents (a)=sample mass (g), (b)=extraction solvent, (c)=Sonication time (min), (d)=Vortex time (s), (e)=Centrifuge speed (RPM), (f)=Centrifuge time (min)

**Text S4**

Optimisation of the Liquid-Liquid Extraction (LLE) and Solid-Liquid Extraction (SLE) procedure

Sample preparation: Each sample volume had spiked de-ionised water (QC) and unspiked de-ionised water (blanks) as procedural blanks for leachate samples. Sediment samples also had spiked sodium sulphate (Na_2_SO_4_) (QC) and unspiked Na_2_SO_4_ as procedural blanks. Samples from Hatherly and Marie Louis were transferred into 50 mL PP falcon tube. Hatherly and Marie Louis landfill sites are the largest and busisest landfill sites in Tshwane and Johannesburg respectively, and therefore, were chosen for method development as representatives of the matrix in their cities. Landfill leachate and sediment for each site including spiked and unspiked procedural blanks were prepared in duplicates.

Spiking procedure: All samples except the procedural blanks were spiked with an OPFR internal standard (IS) mix (dTPP, dTBP, dTPrP ^13^C_18_MTPP) of 25 ng and allowed to stand overnight to equilibrate with the sample matrix. The following day, the samples were then subject to LLE and SLE extraction procedure by Sibiya, et al. ^2^. This method was ammended as the need arose using green solvents.

Leachate extraction procedure: After the spiking of leachate samples and blanks, 10 mL of organic solvent and synthesised DES was added to the tested samples separately, capped and ultra-sonicated for 10 min, vortexed for 2 min, then centrifuged at 3500 RPM for 10 min; these conditions were used to kickstart the extraction based on Sibiya, et al. ^2^. Similarly, hexane: acetone (3:1) was used to initiate the extraction process based on its effiency to extract OPFRs, as reported by Sibiya, et al. ^2^. DES-3 was selected to initiate the optimisation process due to its less viscous nature and ability to efficiently extract polycylic aromatic hydrocarbons (PAHs) ^3,4^. A homogenous solution was formed instead of separate layers after centrifuging for DES, but two layers were formed for organic solvents. Therefore, the DES solution was transferred into 250 mL round bottom flasks and the water content in the solution evaporated using vacuum rotary evaporator (RotaVapor R-210, BÜCHI Labortecnik AG, Switzerland) to about 10 mL, and this gave rise to the non-volatile DES. Five millilitres of hexane was added to the DES in the 250 mL round bottom flask. It was vortexed for 2 min, transferred into 50 mL PP clean falcon tubes and centrifuged at 3500 RPM for 10 min. The hexane supernatants obtained from DES and organic solvent extracts were transferred into pre-cleaned glass tubes and then concentrated under gentle nitrogen stream to dryness and then reconstituded with 1 mL of hexane. Thereafter, the samples were subjected to column clean-up procedures.

Sediment extraction procedure: Sediment samples and blanks were spiked and 5 mL of synthesised DES-3 and hexane: acetone (3:1) solvent was added to the PP falcon tubes with samples. The falcon tubes were then capped and ultra-sonicated for 5 min, vortexed for 2 min, then centrifuged at 3500 RPM for 5 min. The supernatants were transferred into pre-cleaned glass tubes. The procedure was repeated twice on the same sample, and blank resulting in the final volume of the supernatant being 10 mL when combined. The next step for DES is solvent exchange, whereby 5 mL of hexane was added into each of the resultant 10 mL supernatant obtained from the extraction of DES and vortexed for 2 min. The contents were added into separate 50 mL PP falcon tubes and centrifuged for 5 min at 3500 RPM and the hexane supernatant was removed and placed into a new set of pre-cleaned glass test tubes;for validation and application to real samples, the conditions used for solvent exchange were similar to optimum parameters obtained. The hexane supernatants for DES and organic solvent extraction were transferred into pre-cleaned glass tubes and then concentrated under gentle nitrogen stream to dryness and then reconstituded with 1 mL of hexane. Thereafter, the samples were subject to a column clean-up procedure.

Sample clean-up procedure: The 1 mL of hexane extracts were cleaned through elution onto a Florisil cartridge pre-conditioned with 6 mL of EtAc and 6 mL of hexane. Fractionation was achieved with 12 mL of an hexane/DCM mixture (4:1, v/v) (Discarded fraction (DF) ,F1) and 10 mL of EtAc (leachate fraction (LF), F2, containing the target compounds collected. F2 was evaporated to near dryness, solubilised in a mixture of 950 μL MeOH:EtAc (8:2) and 50 μL RS (50 pg/μL), and finally transferred into amber vials for injection into the LC-MS/MS. For sediment samples, Sediment fraction (F2) was then filtered through a 0.22 µm filter then evaporated to near dryness, solubilised in a mixture of 950 μL MeOH:EtAc (8:2) and 50 μL reconstitution internal standard (RIS) (50 pg/μL), the mix was in turn diluted 50 times with MeOH:EtAc (8:2) to 1 mL amber vials then injected into the LC-MS/MS. Each sample vial was injected twice and the mean of the injections recorded. Supplementary Fig S5 is a schematic representation of the sample clean–up procedure followed in this study.

After the optimum sample volume and mass were obtained, they were then used and the optimisation of organic solvent and DES commenced while the extraction conditions remained the same. When the optimum extracting solvents were obtained the sonication time variables were chnaged while other conditions remained the same, the process was repeated till optimum variables for each extraction parameter was obtained.


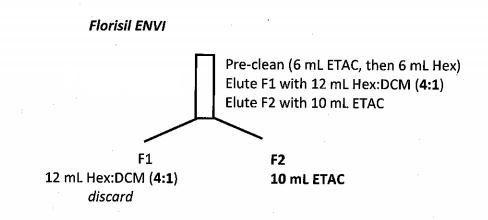


**Figure S5** Schematic representation of the sample clean–up procedure

**Text S5:**

Figure S6 shows the extraction flow chart of targeted OPFRs in leachate using green DES solvent for extraction. After the spiking of samples and blanks, 5 mL of synthesised DES-3 was added to the samples, capped and ultra-sonicated for 5 min, vortexed for 1 min, then centrifuged A homogenous solution was formed after centrifuging. Therefore, the solution was transferred into 250 mL round bottom flasks and water content evaporated using the vacuum rotary evaporator (RotaVapor R-210, BÜCHI Labortecnik AG, Switzerland) to about 5 mL, which is the non-volatile DES.

Five millilitres of hexane was added to the DES in the 250 mL round bottom flask. It was vortexed for 1 min, transferred into 50 mL PP clean falcon tubes and centrifuged at 3500 RPM for 10 min. The hexane supernatant was transferred into pre-cleaned glass tubes and then concentrated under gentle nitrogen stream to dryness and then reconstituded with 1 mL of hexane. Thereafter, the samples were subject to a column clean-up procedure as described in Text S4.


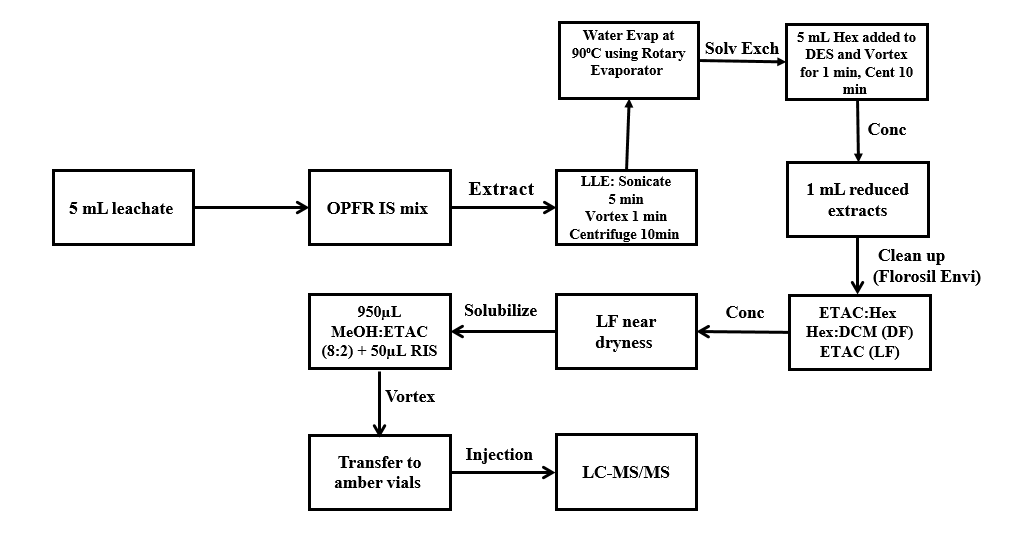


**Figure S6** Extraction flow chart of targeted OPFRs in leachate using DES-3 for extraction.

**Text S6:**

Figure S7 is the extraction flow chart of targeted OPFRs in sediment using green DES solvent. Five millilitres (5 mL) of synthesised DES-1 was added to the falcon tubes with spiked sediment samples, the falcon tubes were capped and ultra-sonicated for 10 min, vortexed for 1 min, and then centrifuged at 2500 RPM for 5 min. The supernatant was transferred into a pre-cleaned glass tubes. The procedure was repeated twice resulting in the final volume of the supernatant of each sample extract being 10 mL when combined.

Five milliliters of hexane was added into each of the resultant 10 mL supernatant obtained from the extraction, this was then vortexed for 1 min. The contents were transferred into separate 50 mL PP falcon tubes and centrifuged for 5 min at 2500 RPM and the hexane supernatant was removed and put into a new set of pre-cleaned glass test tubes. The supernatant was then concentrated under a gentle nitrogen stream to dryness and then reconstituded with 1 mL of hexane. Thereafter, the samples were subject to a column clean-up procedure as described in Text S4.


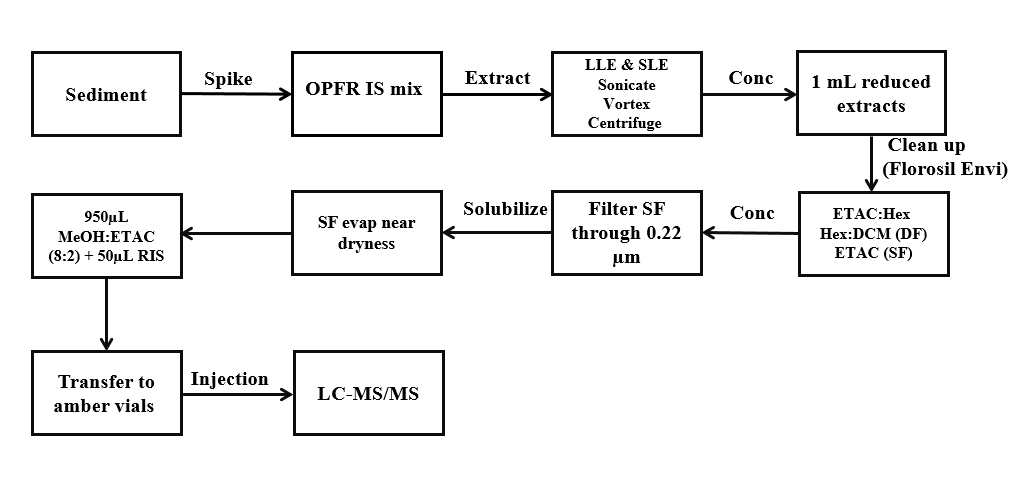


**Figure S7** Extraction flow chart of targeted OPFRs in sediment using DES-1 for extraction

**Text S7:**

The extraction of targeted OPFRs in leachate and sediment using hexane: 5 mL organic solvent (hexane) was added to the falcon tubes with the spiked samples. Leachate samples (5mL) were sonicated for 20 min and sediment samples (0.3 g) for 10 min after being spiked, then they were vortexed for 1 min. Thereafter, leachate was centrifuged at 3500 RPM for 20 min and sediment was centrifuged at 2500 RPM for 5 min. The supernatant of the sample was transferred into a pre-cleaned glass tubes. The procedure was repeated twice. The combined supernatant (10 mL) of each sample was then concentrated under gentle nitrogen stream to dryness, and reconstitued with 1 mL hexane. Thereafter, the samples were subject to a column clean-up procedure as described in Text S4 until samples were injected in the LC-MS/MS.


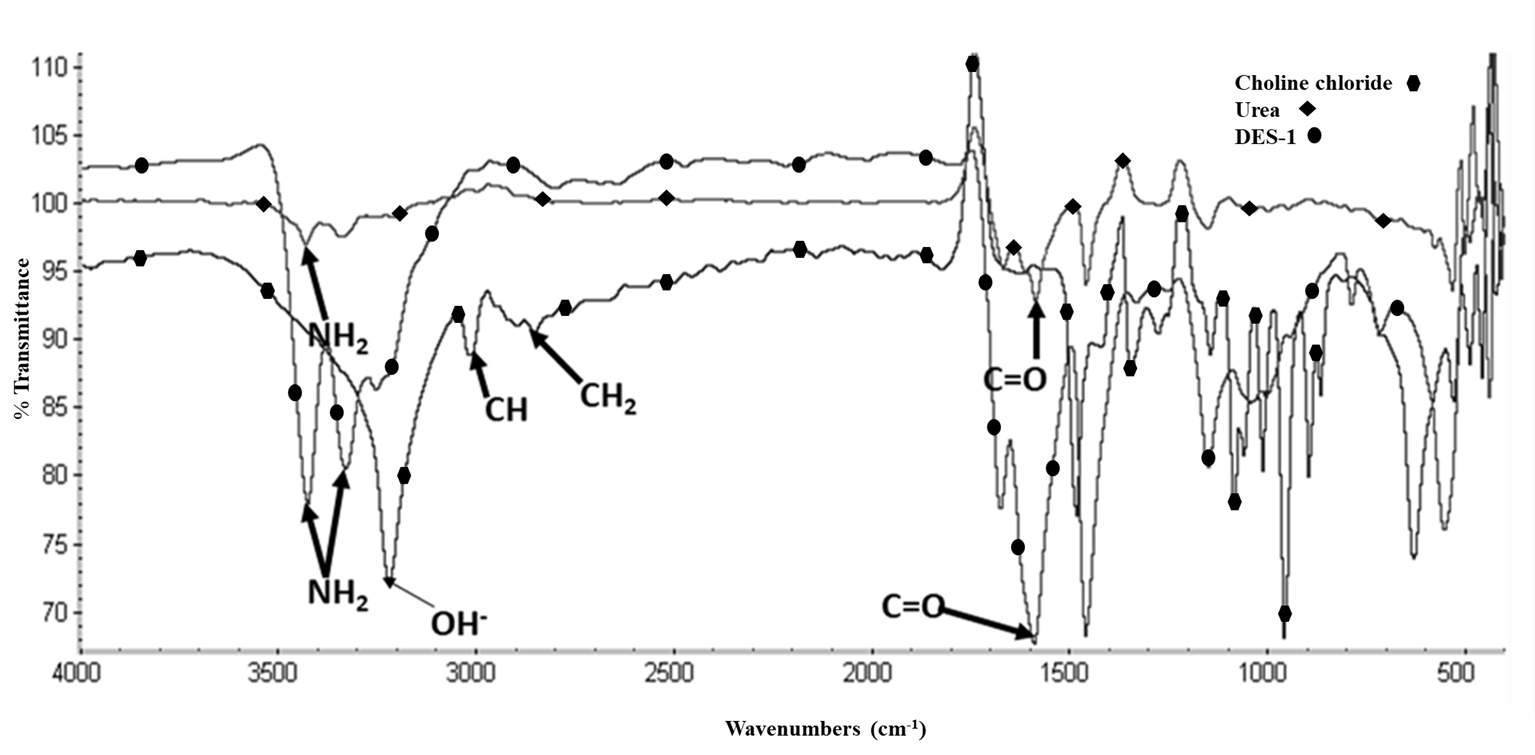


**Figure S8** FTIR spectra of Choline chloride, Urea and Choline chloride Urea DES


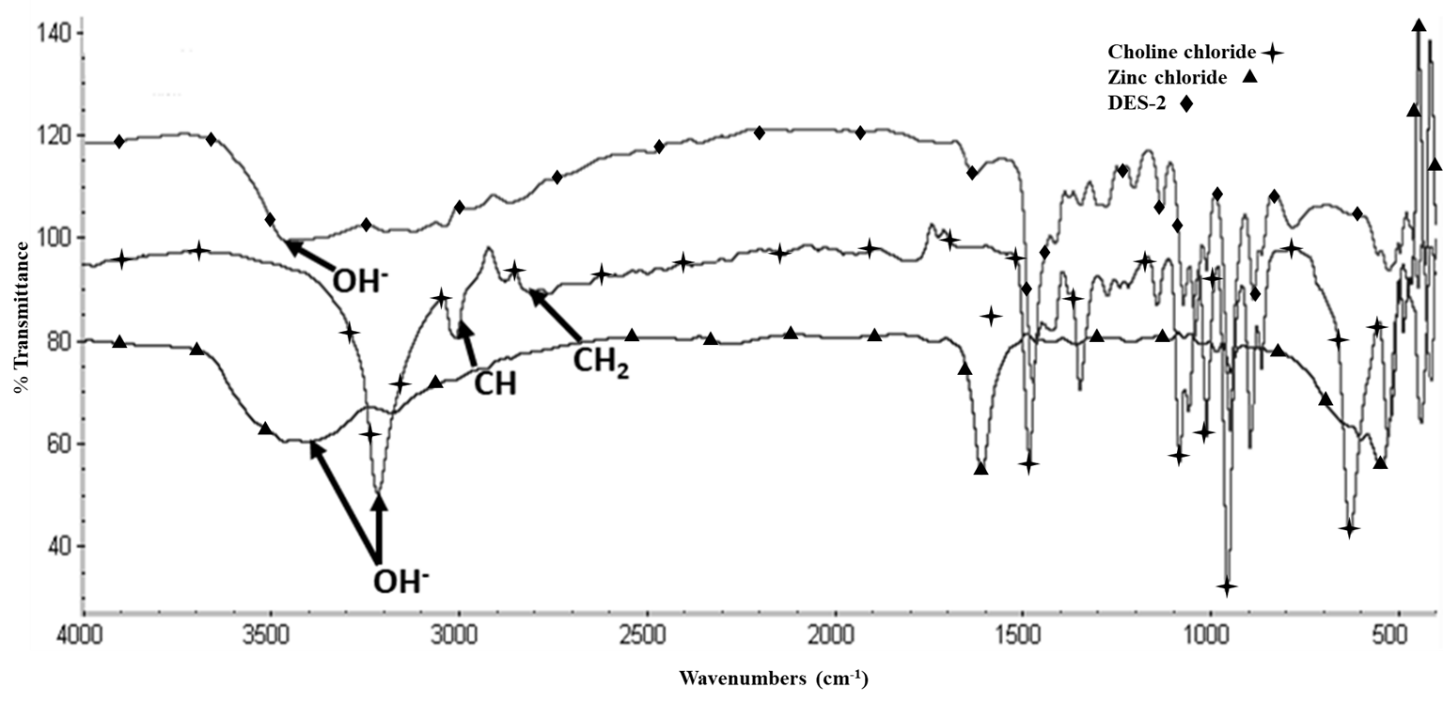


**Figure S9** FTIR spectra of Choline chloride, Zinc chloride and Choline chloride Zinc chloride DES


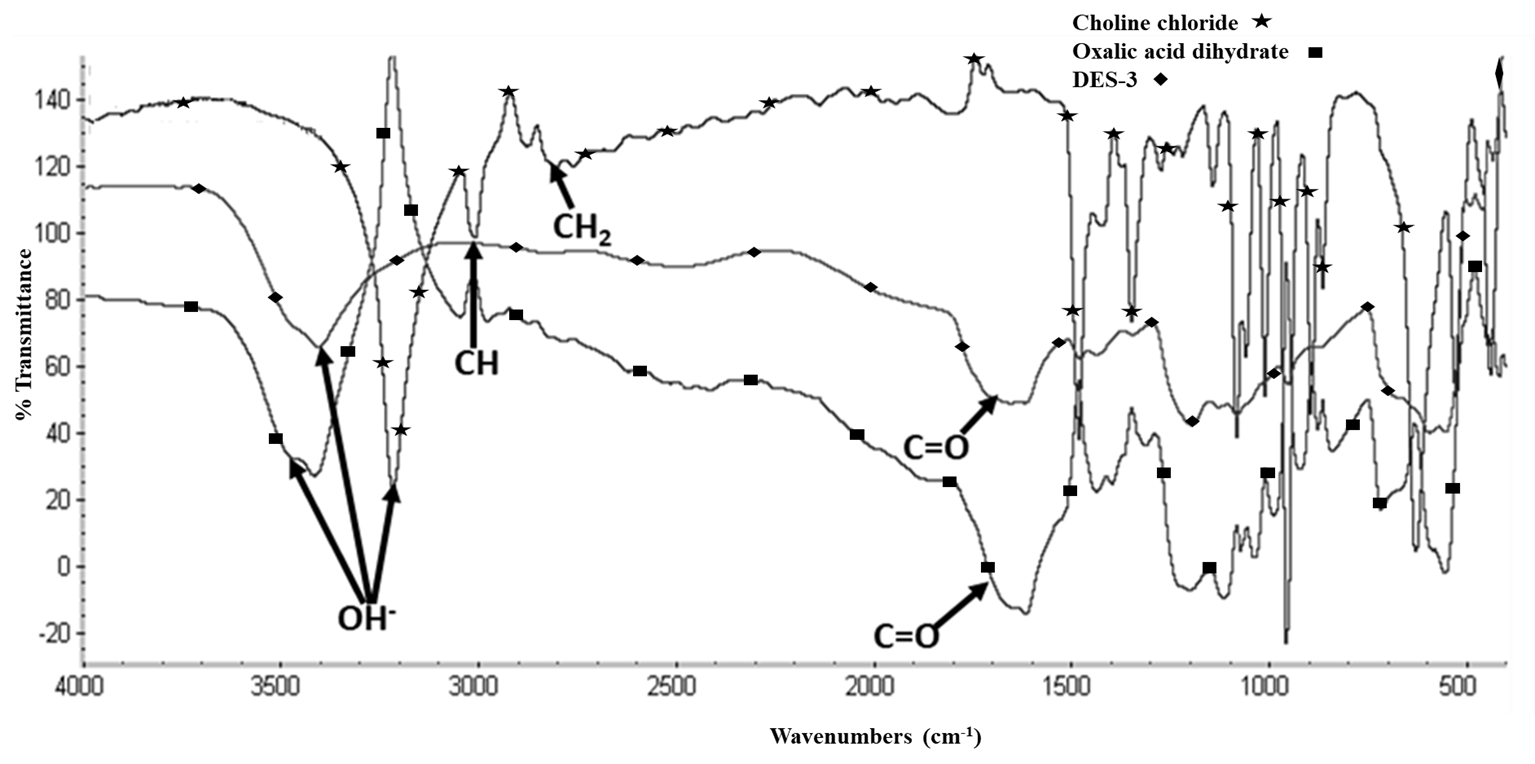


**Figure S10** FTIR spectra of Choline chloride, Oxalic acid dihydrate and Choline chloride Oxalic acid. Dehydrate (1:1)


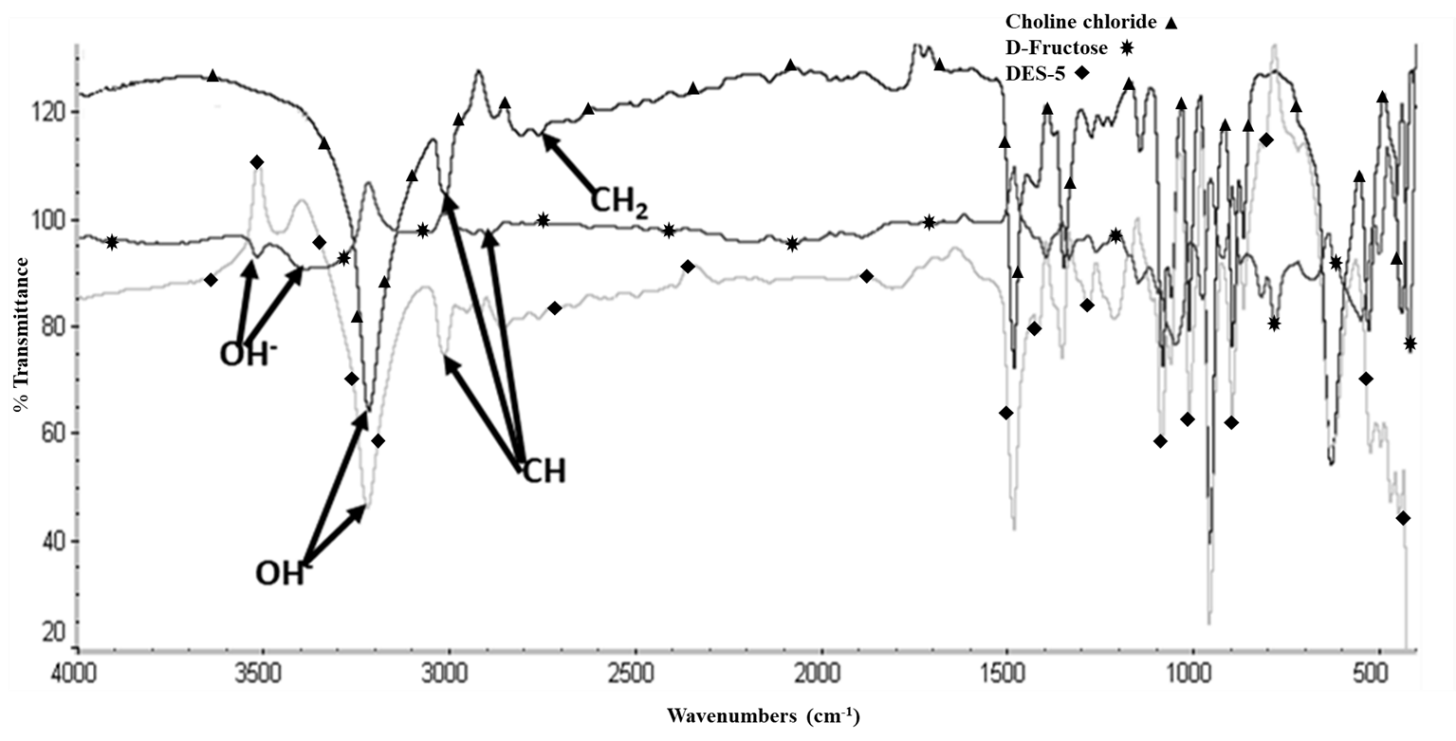


**Figure S11** FTIR spectra of Choline chloride, D-Fructose and Choline chloride D-Fructose DES


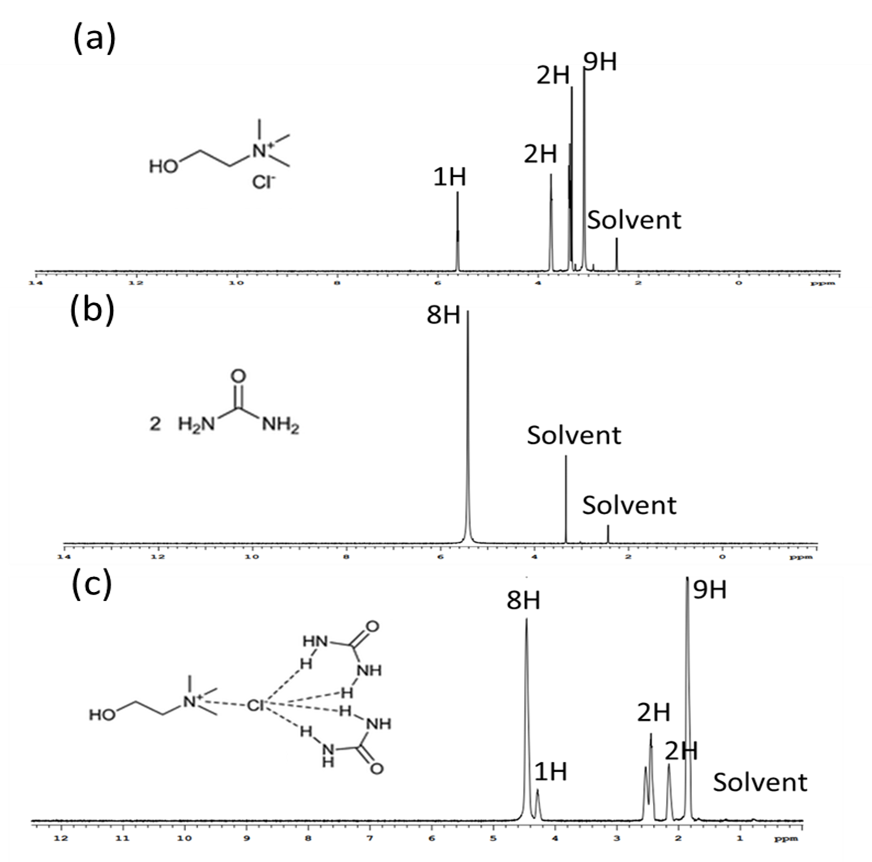


**Figure S12** ^1^H NMR spectra of (a) choline chloride, (b) urea and (c) choline chloride urea DES


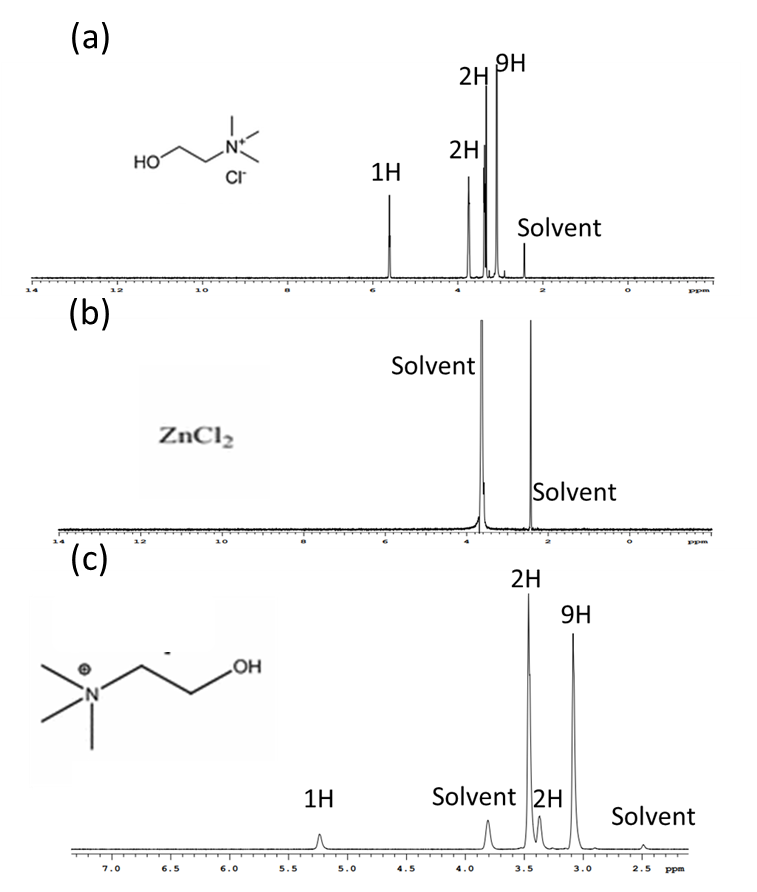


**Figure S13** ^1^H NMR spectra of (a) choline chloride, (b) zinc chloride and (c) choline chloride zinc chloride DES


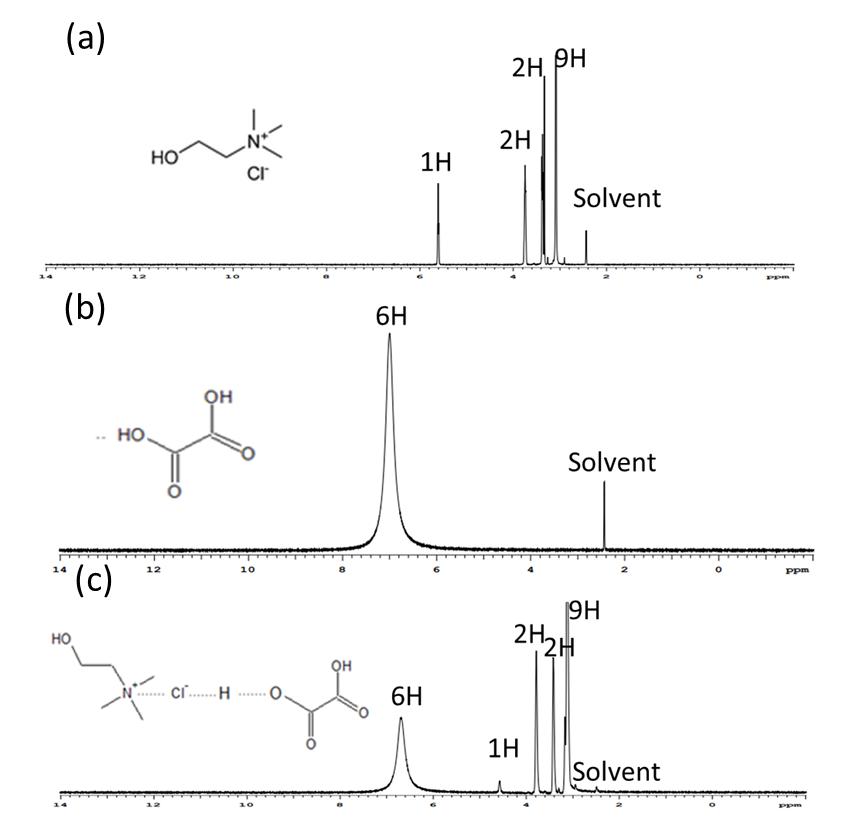


**Figure S14** ^1^H NMR spectra of (a) choline chloride, (b) oxalic acid dihydrate and (c) choline chloride oxalic acid dihydrate


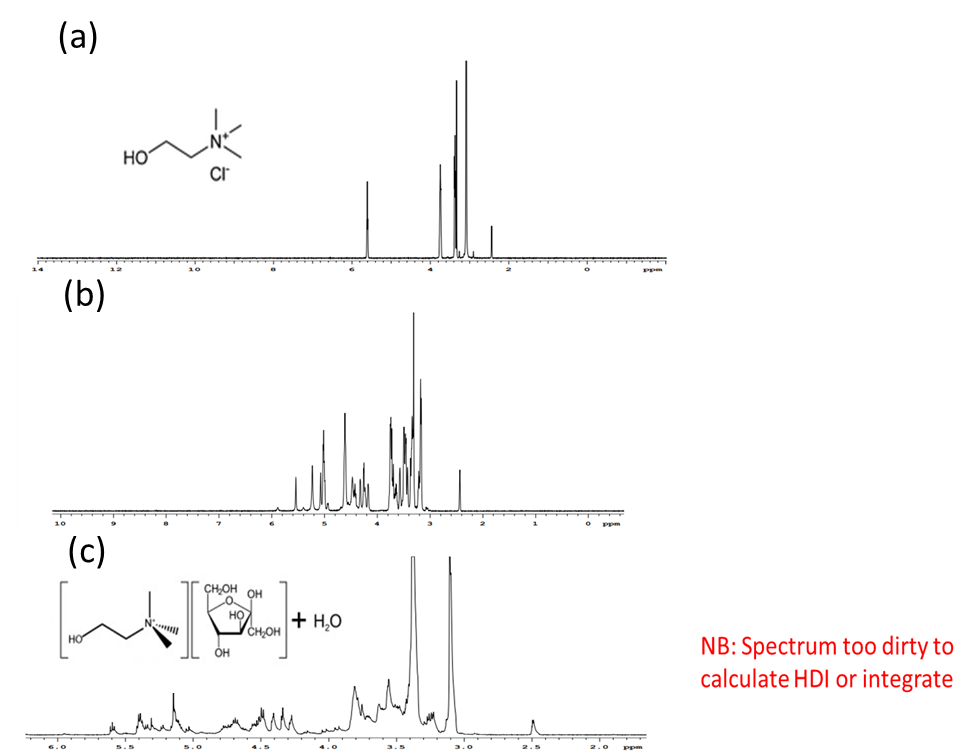


**Figure S15** ^1^H NMR spectra of (a) choline chloride, (b) D-fructose and (c) choline chloride D-fructose

**Table S4** Certified and measured (n=2) concentrations (ng/g) of EDF-2525 contaminated natural matrix reference material extracted using hexane.

| **Congener** | **Certified** | **Measured** | **Recovery (%)** |
| --- | --- | --- | --- |
| TPP | 1.39 ± 1.00 | 1.65 ± 0.56 | 84.7 |
| TOTP | 0.29 ± 0.18 | 0.32 ± 0.26 | 90.6 |
| TMTP | 0.23 ± 0.15 | 0.24 ± 0.19 | 95.8 |
| TPTP | 0.12 ± 0.15 | 0.11 ± 0.08 | 109 |
| T35DMPP | 0.13 ± 0.50 | 0.16 ± 0.12 | 81.2 |
| T21PPP | 1.95 ± 0.34 | 2.00 ± 2.50 | 97.5 |
| TEP | 3.39 ± 0.85 | 3.40 ± 2.32 | 99.7 |
| TPrP | 4.47 ± 1.85 | 4.60 ± 2.93 | 97.1 |
| TBEP | 4.28 ± 0.99 | 4.50 ± 2.98 | 95.1 |
| TDBPP | 4.24 ± 0.15 | 4.20 ± 1.89 | 100 |
| EHDP | 2.5 ± 1.82 | 2.70 ± 1.97 | 92.5 |
| TEHP | 0.91 ± 0.50 | 0.92 ± 1.60 | 98.9 |
| TCEP | 2.89 ± 1.23 | 2.90 ± 0.60 | 99.6 |
| TCPP | 2.38 ± 0.18 | 2.40 ± 1.50 | 99.2 |
| TDCPP | 3.50 ± 1.50 | 03.7 ± 1.00 | 94.6 |
| TBP | 4.60 ± 3.50 | 3.65 ± 2.86 | 95.8 |
| TPP | 3.50 ± 2.60 | 3.70 ± 2.98 | 92.1. |

**Table S5** Certified and measured (n=2) concentrations (ng/g) of EDF-2525 contaminated natural matrix reference material extracted using DES-1

| **Congener** | **Certified** | **Measured** | **Recovery (%)** |
| --- | --- | --- | --- |
| TPP | 1.39 ± 1.00 | 1.50 ± 0.56 | 92.6 |
| TOTP | 0.29 ± 0.18 | 0.34 ± 0.26 | 85.2 |
| TMTP | 0.23 ± 0.15 | 0.20 ± 0.19 | 115 |
| TPTP | 0.12 ± 0.15 | 0.13 ± 0.08 | 92.3 |
| T35DMPP | 0.13 ± 0.50 | 0.14 ± 0.12 | 92.8 |
| T21PPP | 1.95 ± 0.34 | 2.20 ± 1.50 | 99.2 |
| TEP | 3.39 ± 0.85 | 3.40 ± 2.32 | 99.7 |
| TPrP | 4.47 ± 1.85 | 4.50 ± 1.93 | 99.3 |
| TBEP | 4.28 ± 0.99 | 4.00 ± 2.98 | 107 |
| TDBPP | 4.24 ± 0.15 | 4.10 ± 2.89 | 103 |
| EHDP | 2.5 ± 1.82 | 2.32 ± 1.97 | 107 |
| TEHP | 0.91 ± 0.50 | 0.90 ± 0.06 | 101 |
| TCEP | 2.89 ± 1.23 | 3.00 ± 2.60 | 96.3 |
| TCPP | 2.38 ± 0.18 | 2.40 ± 1.50 | 99.2 |
| TDCPP | 3.50 ± 1.50 | 3.00 ± 1.00 | 116 |
| TBP | 4.60 ± 3.50 | 3.90 ± 1.86 | 118 |
| TPP | 3.50 ± 2.60 | 3.00 ± 2.08 | 116 |

**Text S8**

QA/QC

Determination of Limits Of Detection (LODs) and Limits of Quantification (LOQs)

This study evaluated both statistical and empirical methods for LOD and LOQ calculations, for targeted compounds (OPFRs) using LC-MS/MS. A procedural blank was analysed every ten samples to check for laboratory contamination. Statistically, if analytes were detected in the procedural blanks, the blank mean concentrations were subtracted from the values found in the samples. The mean blank value as well as the standard deviation were then calculated and the LOD was determined by:

𝐿𝑂𝐷 = 𝑚𝑒𝑎𝑛 𝑏𝑙𝑎𝑛𝑘 𝑣𝑎𝑙𝑢𝑒 + (3 × 𝑆𝐷)

If analytes were not detected in the blanks, the LODs and LOQs were calculated empirically based on the signal/noise ratio for a standard of known concentration. The LOD was three times the signal to noise ratio (S/N 3:1) and for LOQ it was ten times the signal to noise ratio (S/N 10:1) of the lowest calibration level.

**Text S9**

Comparison of OPFR recoveries by other extraction methods

In aqueous media, the following recoveries have been reported, 67–125% ^5-8^ using LLE. Similarly, 65.5–134% has been reported using solid phase extraction (SPE) ^9-17^; 86–106% with dispersive liquid-liquid micro extraction (DLLME) ^18^; 80.5–112% ^19^ using microwave assisted headspace solid phase micro extraction (MA-HS-SPME); 80.5–112% for headspace solid phase micro extraction (HS-SPME) ^20^ and 93–102% ^21^ with Stir bar sorptive extraction (SBSE). The recoveries in the present study have comparable values to the aforementioned values.

Lee, et al. ^6^ and Woudneh, et al. ^8^ reported 67-125% recoveries for OPFRs in solid media using Soxhlet extraction. Others reported percentage recoveries ranging from 52.9-128% for sonication ^22-24^; 67–103% using pressurized liquid extraction (PLE) ^25^, 78–105% with microwave extraction ^26^ and 59.4–114% by shaking ^27^.

**Figure S16** General waste (tonnages) disposed in June and the whole year in 2019 ^28^

**Table S6** Comparison of the range of OPFR concentrations in landfill leachate and sediment from studies around the globe, extracted using organic solvents.

| **Compound** | **Matrix** | **Location** | **Extraction solvent** | **Range of total OPFRs, leachate (ng/L) and sediment (ng/g)** | **Reference** |
| --- | --- | --- | --- | --- | --- |
| TnPP, TnBP, TCEP, TCIPP TDCIPP, TPHP, EHDHP, TEHP, TOTP, TCP, T2IPPP, TPTP, T35DMPP, TBPP | Landfill leachate | Gauteng, South Africa | Hex:Ace (3:1)  More non-polar | 556-17200 | Sibiya, et al. ^2^ |
| TNBP | Landfill leachate | Sweden | Dichloromethane (polar) | <2−7 | Öman and Hynning ^29^ |
| TBOEP, TCEP, TCIPP, TDCIPP, TPHP, TEP, TPP, TIBP | Landfill leachate | Central/North eastern Japan | Dichloromethane (polar) | 4.1−5430 | Yasuhara ^30^ |
| TBOEP, TCEP, TCIPP, TDCIPP, TPHP, TEP, TPP, TIBP, TNBP | Landfill leachate | Japan | Dichloromethane (polar) | 0.14−153 | Kawagoshi, et al. ^31^ |
| TBOEP, TCEP, TCIPP, TDCIPP, TPHP, TEP, TPP, TIBP, TNBP | Landfill leachate | Japan | Dichloromethane (polar) | 0.69−67.2 | Yasuhara ^30^ |
| TBOEP, TCEP, TCIPP, TDCIPP, TPHP, TEP, TPP, TIBP, TNBP | Landfill leachate | Sweden | - | 67−174 | Paxéus ^32^ |
| TBOEP, TCEP, TCIPP, TDCIPP, TPHP, TEP, TPP, TIBP, TNBP | Landfill leachate | Germany | n-pentane, dichloromethane (1:1)  More polar | 239−693 | Schwarzbauer, et al. ^33^ |
| TBOEP, TCEP, TCIPP, TDCIPP, TPHP, TEP, TPP, TIBP, TNBP | Landfill leachate | USA | Dichloromethane (polar) | 3.50−5.12 | Andrews, et al. ^34^ |
| TMP, TEP, TCEP, TCPP, TDCP, TBEP, TnBP, TiBP, TPhP, TPrP, TCrP, CDPP, EHDPP and TEHP | Landfill leachate | Beijing, China | Acetonitrile (polar) | 16.2−44.8 | Gao, et al. ^27^ |
| TEP, TPrP, TBP, TCEP, TCPP, TDCP, TBEP, TPP, TCP | Landfill leachate | Guangdong Province, China | Ethyl acetate and dichloromethane (1:1) (polar) | 0.00702 - 4.81 | Deng, et al. ^35^ |
| TEP, TIBP, TNBP, [TBOEP](https://www.sciencedirect.com/topics/earth-and-planetary-sciences/transparent-exopolymer-particle), TCEP, TCIPP, and TMPP, TDCIPP and [TPHP](https://www.sciencedirect.com/topics/earth-and-planetary-sciences/triphenyls) | Landfill leachate | China | 25% Dichloromethane and acetonitrile  (polar) | 29.0−437 | Qi, et al. ^36^ |
| TOTP, TPP, TMTP, TCEP,  TCPP, TPTP, T35DMPP,  T21PPP, TBEP, TEHP, EHDP, TPrP, TEP, TDCPP | Landfill leachate | Gauteng, South Africa. | Hexane | 5.90 -981 | This study |
| TOTP, TPP, TMTP, TCEP,  TCPP, TPTP, T35DMPP,  T21PPP, TBEP, TEHP, EHDP, TPrP, TEP, TDCPP | Landfill leachate | Gauteng, South Africa. | DES-3 | 510 – 1433 | This study |
| TDCIPP, TIBP, TEP, TPP, TNBP, TCEP, TCIPP, (TBOEP, TPHP, EHDPP, TEHP, TMPP | Sediment from multi-waste recycling area | Tianjin, China | Hex: Ace (1:1) | <MDL – 548 | Wang, et al. ^24^ |
| TnPP, TnBP, TCEP, TCIPP TDCIPP, TPHP, EHDHP, TEHP, TOTP, TCP, T2IPPP, TPTP, T35DMPP, TBPP | Landfill Sediment | South Africa | Hex:Ace (3:1) | <LOQ - 741 | Sibiya, et al. ^2^ |
| TOTP, TPP, TMTP, TCEP,  TCPP, TPTP, T35DMPP,  T21PPP, TBEP, TEHP, EHDP, TPrP, TEP, TDCPP | Landfill Sediment | South Africa | Hexane | <LOQ - 298 | This study |
| TOTP, TPP, TMTP, TCEP,  TCPP, TPTP, T35DMPP,  T21PPP, TBEP, TEHP, EHDP, TPrP, TDCPP | Landfill Sediment | South Africa | DES-1 | 725 - 1224 | This study |

**References**

1 Brandsma, S. H., de Boer, J., Cofino, W. P., Covaci, A. & Leonards, P. E. G. Organophosphorus flame-retardant and plasticizer analysis, including recommendations from the first worldwide interlaboratory study. *Trac-Trends in Analytical Chemistry* **43**, 217-228, doi:<https://doi.org/10.1016/j.trac.2012.12.004> (2013).

2 Sibiya, I. *et al.* Targeted and non-target screening of persistent organic pollutants and organophosphorus flame retardants in leachate and sediment from landfill sites in Gauteng Province, South Africa. *Science of The Total Environment* **653**, 1231-1239, doi:<https://doi.org/10.1016/j.scitotenv.2018.10.356> (2019).

3 Mbous, Y. P. *et al.* Applications of deep eutectic solvents in biotechnology and bioengineering—Promises and challenges. *Biotechnology advances* (2016).

4 Płotka-Wasylka, J., Rutkowska, M., Owczarek, K., Tobiszewski, M. & Namieśnik, J. Extraction with environmentally friendly solvents. *TrAC Trends in Analytical Chemistry* (2017).

5 Martínez-Carballo, E., González-Barreiro, C., Sitka, A., Scharf, S. & Gans, O. Determination of selected organophosphate esters in the aquatic environment of Austria. *Science of The Total Environment* **388**, 290-299, doi:<https://doi.org/10.1016/j.scitotenv.2007.08.005> (2007).

6 Lee, S., Cho, H.-J., Choi, W. & Moon, H.-B. Organophosphate flame retardants (OPFRs) in water and sediment: Occurrence, distribution, and hotspots of contamination of Lake Shihwa, Korea. *Marine Pollution Bulletin* **130**, 105-112, doi:<https://doi.org/10.1016/j.marpolbul.2018.03.009> (2018).

7 Lee, S., Jeong, W., Kannan, K. & Moon, H.-B. Occurrence and exposure assessment of organophosphate flame retardants (OPFRs) through the consumption of drinking water in Korea. *Water Research* **103**, 182-188, doi:<https://doi.org/10.1016/j.watres.2016.07.034> (2016).

8 Woudneh, M. B. *et al.* Quantitative determination of 13 organophosphorous flame retardants and plasticizers in a wastewater treatment system by high performance liquid chromatography tandem mass spectrometry. *Journal of Chromatography A* **1400**, 149-155, doi:<https://doi.org/10.1016/j.chroma.2015.04.026> (2015).

9 Rodríguez, I. *et al.* Suitability of solid-phase microextraction for the determination of organophosphate flame retardants and plasticizers in water samples. *Journal of Chromatography A* **1108**, 158-165, doi:<https://doi.org/10.1016/j.chroma.2006.01.008> (2006).

10 García-López, M., Rodríguez, I. & Cela, R. Mixed-mode solid-phase extraction followed by liquid chromatography–tandem mass spectrometry for the determination of tri- and di-substituted organophosphorus species in water samples. *Journal of Chromatography A* **1217**, 1476-1484, doi:<https://doi.org/10.1016/j.chroma.2009.12.067> (2010).

11 Gustavsson, J. *et al.* Screening of organic flame retardants in Swedish river water. *Science of The Total Environment* **625**, 1046-1055, doi:<https://doi.org/10.1016/j.scitotenv.2017.12.281> (2018).

12 Lorenzo, M., Campo, J. & Picó, Y. Ultra-high-pressure liquid chromatography tandem mass spectrometry method for the determination of 9 organophosphate flame retardants in water samples. *MethodsX* **3**, 343-349, doi:<https://doi.org/10.1016/j.mex.2016.04.006> (2016).

13 Wang, X.-w., Liu, J.-f. & Yin, Y.-g. Development of an ultra-high-performance liquid chromatography–tandem mass spectrometry method for high throughput determination of organophosphorus flame retardants in environmental water. *Journal of Chromatography A* **1218**, 6705-6711, doi:<https://doi.org/10.1016/j.chroma.2011.07.067> (2011).

14 Aznar-Alemany, Ò. *et al.* Halogenated and organophosphorus flame retardants in European aquaculture samples. *Science of The Total Environment* **612**, 492-500, doi:<https://doi.org/10.1016/j.scitotenv.2017.08.199> (2018).

15 Bollmann, U. E., Moller, A., Xie, Z., Ebinghaus, R. & Einax, J. W. Occurrence and fate of organophosphorus flame retardants and plasticizers in coastal and marine surface waters. *Water Res* **46**, 531-538, doi:<https://doi.org/10.1016/j.watres.2011.11.028> (2012).

16 Regnery, J. & Püttmann, W. Occurrence and fate of organophosphorus flame retardants and plasticizers in urban and remote surface waters in Germany. *Water research* **44**, 4097-4104, doi:<https://doi.org/10.1016/j.watres.2010.05.024> (2010).

17 Shi, Y. *et al.* Occurrence, distribution and seasonal variation of organophosphate flame retardants and plasticizers in urban surface water in Beijing, China. *Environmental Pollution* **209**, 1-10, doi:<https://doi.org/10.1016/j.envpol.2015.11.008> (2016).

18 García-López, M., Rodríguez, I. & Cela, R. Development of a dispersive liquid–liquid microextraction method for organophosphorus flame retardants and plasticizers determination in water samples. *Journal of Chromatography A* **1166**, 9-15, doi:<https://doi.org/10.1016/j.chroma.2007.08.006> (2007).

19 Shahbodaghi, M., Faraji, H., Shahbaazi, H. & Shabani, M. Sustainable and green microextraction of organophosphorus flame retardants by a novel phosphonium-based deep eutectic solvent. *Journal of Separation Science* **43**, 452-461, doi:<https://doi.org/10.1002/jssc.201900504> (2020).

20 Jin, X. *et al.* Risk assessment of organochlorine pesticides in drinking water source of the Yangtze River. *Ecotoxicology and Environmental Safety* **182**, 109390, doi:<https://doi.org/10.1016/j.ecoenv.2019.109390> (2019).

21 Bridoux, M. C., Malandain, H., Leprince, F., Progent, F. & Machuron-Mandard, X. Quantitative analysis of phosphoric acid esters in aqueous samples by isotope dilution stir-bar sorptive extraction combined with direct analysis in real time (DART)-Orbitrap mass spectrometry. *Analytica Chimica Acta* **869**, 1-10, doi:<https://doi.org/10.1016/j.aca.2015.01.010> (2015).

22 Cristale, J., Vázquez, A. G., Barata, C. & Lacorte, S. Priority and emerging flame retardants in rivers: occurrence in water and sediment, Daphnia magna toxicity and risk assessment. *Environment international* **59**, 232-243, doi:<https://doi.org/10.1016/j.envint.2013.06.011> (2013).

23 Pang, L. *et al.* Occurrence, distribution, and potential affecting factors of organophosphate flame retardants in sewage sludge of wastewater treatment plants in Henan Province, Central China. *Chemosphere* **152**, 245-251, doi:<https://doi.org/10.1016/j.chemosphere.2016.02.104> (2016).

24 Wang, Y. *et al.* Occurrence and distribution of organophosphate flame retardants (OPFRs) in soil and outdoor settled dust from a multi-waste recycling area in China. *Science of The Total Environment* **625**, 1056-1064, doi:<https://doi.org/10.1016/j.scitotenv.2018.01.013> (2018).

25 Giulivo, M. *et al.* Occurrence of halogenated and organophosphate flame retardants in sediment and fish samples from three European river basins. *Science of The Total Environment* **586**, 782-791, doi:<https://doi.org/10.1016/j.scitotenv.2017.02.056> (2017).

26 García-López, M., Rodríguez, I., Cela, R., Kroening, K. K. & Caruso, J. A. Determination of organophosphate flame retardants and plasticizers in sediment samples using microwave-assisted extraction and gas chromatography with inductively coupled plasma mass spectrometry. *Talanta* **79**, 824-829, doi:<https://doi.org/10.1016/j.talanta.2009.05.006> (2009).

27 Gao, L., Shi, Y., Li, W., Liu, J. & Cai, Y. Occurrence and distribution of organophosphate triesters and diesters in sludge from sewage treatment plants of Beijing, China. *Science of The Total Environment* **544**, 143-149, doi:<https://doi.org/10.1016/j.scitotenv.2015.11.094> (2016).

28 GWIS. *The Gauteng Waste Information System (GWIS) is intended to provide the public, business, industry and government with access to information on the management of waste within the Gauteng Province, by capturing routine data on the tonnes of waste transported, treated, landfilled and recycled in the Province on a monthly and annual basis.*, <<https://gwis.gpg.gov.za/reports>> (2021).

29 Öman, C. & Hynning, P.-Å. Identification of organic compounds in municipal landfill leachates. *Environmental Pollution* **80**, 265-271, doi:<https://doi.org/10.1016/0269-7491(93)90047-R> (1993).

30 Yasuhara, A. Chemical components in leachates from hazardous wastes landfills in Japan. *Toxicological & Environmental Chemistry* **51**, 113-120, doi:<https://doi.org/10.1080/02772249509358229> (1995).

31 Kawagoshi, Y., Fukunaga, I. & Itoh, H. Distribution of organophosphoric acid triesters between water and sediment at a sea-based solid waste disposal site. *Journal of Material Cycles and Waste Management* **1**, 53-61, doi:<https://doi.org/s10163-999-0005-6> (1999).

32 Paxéus, N. Organic compounds in municipal landfill leachates. *Water Science and Technology* **42**, 323-333, doi:<https://doi.org/10.2166/wst.2000.0585> (2000).

33 Schwarzbauer, J., Heim, S., Brinker, S. & Littke, R. Occurrence and alteration of organic contaminants in seepage and leakage water from a waste deposit landfill. *Water Research* **36**, 2275-2287, doi:<https://doi.org/10.1016/S0043-1354(01)00452-3> (2002).

34 Andrews, W. J., Masoner, J. R. & Cozzarelli, I. M. Emerging Contaminants at a Closed and an Operating Landfill in Oklahoma. *Groundwater Monitoring & Remediation* **32**, 120-130, doi:<https://doi.org/10.1111/j.1745-6592.2011.01373.x> (2012).

35 Deng, M. *et al.* Organophosphorus flame retardants and heavy metals in municipal landfill leachate treatment system in Guangzhou, China. *Environmental Pollution* **236**, 137-145, doi:<https://doi.org/10.1016/j.envpol.2018.01.042> (2018).

36 Qi, C. *et al.* Organophosphate flame retardants in leachates from six municipal landfills across China. *Chemosphere* **218**, 836-844, doi:<https://doi.org/10.1016/j.chemosphere.2018.11.150> (2019).
